# The 5-Hydroxymethylcytosine Landscape of Prostate Cancer

**DOI:** 10.1158/0008-5472.CAN-22-1123

**Published:** 2022-10-17

**Authors:** Martin Sjöström, Shuang G. Zhao, Samuel Levy, Meng Zhang, Yuhong Ning, Raunak Shrestha, Arian Lundberg, Cameron Herberts, Adam Foye, Rahul Aggarwal, Junjie T. Hua, Haolong Li, Anna Bergamaschi, Corinne Maurice-Dror, Ashutosh Maheshwari, Sujun Chen, Sarah W.S. Ng, Wenbin Ye, Jessica Petricca, Michael Fraser, Lisa Chesner, Marc D. Perry, Thaidy Moreno-Rodriguez, William S. Chen, Joshi J. Alumkal, Jonathan Chou, Alicia K. Morgans, Tomasz M. Beer, George V. Thomas, Martin Gleave, Paul Lloyd, Tierney Phillips, Erin McCarthy, Michael C. Haffner, Amina Zoubeidi, Matti Annala, Robert E. Reiter, Matthew B. Rettig, Owen N. Witte, Lawrence Fong, Rohit Bose, Franklin W. Huang, Jianhua Luo, Anders Bjartell, Joshua M. Lang, Nupam P. Mahajan, Primo N. Lara, Christopher P. Evans, Phuoc T. Tran, Edwin M. Posadas, Chuan He, Xiao-Long Cui, Jiaoti Huang, Wilbert Zwart, Luke A. Gilbert, Christopher A. Maher, Paul C. Boutros, Kim N. Chi, Alan Ashworth, Eric J. Small, Housheng H. He, Alexander W. Wyatt, David A. Quigley, Felix Y. Feng

**Affiliations:** 1 Helen Diller Family Comprehensive Cancer Center, University of California, San Francisco, San Francisco, CA.; 2 Department of Radiation Oncology, University of California, San Francisco, San Francisco, CA.; 3 Division of Oncology, Department of Clinical Sciences Lund, Faculty of Medicine, Lund University, Lund, Sweden.; 4 Department of Human Oncology, University of Wisconsin-Madison, Madison, WI.; 5 William S. Middleton Memorial Veterans’ Hospital, Madison, WI.; 6 Bluestar Genomics Inc., San Diego, CA.; 7 Vancouver Prostate Centre, Department of Urologic Sciences, University of British Columbia, Vancouver, BC, Canada.; 8 Division of Hematology and Oncology, Department of Medicine, University of California, San Francisco, San Francisco, CA.; 9 BC Cancer, Vancouver, BC, Canada.; 10 Department of Medical Biophysics, University of Toronto, Toronto, Ontario, Canada.; 11 Princess Margaret Cancer Centre, University Health Network, Toronto, Ontario, Canada.; 12 Department of Automation, Xiamen University, Xiamen, Fujian, China.; 13 Department of Surgery, University of Toronto, Toronto, Ontario, Canada.; 14 Division of Hematology and Oncology, University of Michigan Rogel Cancer Center, Ann Arbor, MI.; 15 Dana-Farber Cancer Institute, Harvard Medical School, Boston, MA.; 16 Knight Cancer Institute, Oregon Health and Science University, Portland, OR.; 17 Department of Pathology, Oregon Health & Science University, Portland, OR.; 18 Divisions of Human Biology and Clinical Research, Fred Hutchinson Cancer Research Center, Seattle, WA.; 19 University of Washington, Seattle, WA.; 20 Faculty of Medicine and Health Technology, Tampere University and Tays Cancer Centre, Tampere, Finland.; 21 Departments of Medicine, Hematology/Oncology and Urology, David Geffen School of Medicine, University of California Los Angeles, Los Angeles, CA.; 22 Jonsson Comprehensive Cancer Center, University of California Los Angeles, Los Angeles, CA.; 23 VA Greater Los Angeles Healthcare System, Los Angeles, CA.; 24 Department of Microbiology, Immunology, and Molecular Genetics, David Geffen School of Medicine, University of California Los Angeles, Los Angeles, CA.; 25 Department of Urology, University of California, San Francisco, San Francisco, CA.; 26 Department of Anatomy, University of California, San Francisco, San Francisco, CA.; 27 Department of Pathology, University of Pittsburgh, Pittsburgh, PA.; 28 Department of Translational Medicine, Medical Faculty, Lund University, Malmö, Sweden.; 29 Department of Urology, Skåne University Hospital, Malmö, Sweden.; 30 Department of Medicine, University of Wisconsin-Madison, Madison, WI.; 31 Siteman Cancer Center, Washington University, St. Louis, MO.; 32 Division of Hematology Oncology, Department of Internal Medicine, University of California Davis, Sacramento, CA.; 33 Comprehensive Cancer Center, University of California Davis, Sacramento, CA.; 34 Department of Urologic Surgery, University of California Davis, Sacramento, CA.; 35 Department of Radiation Oncology, University of Maryland, College Park, Baltimore, MD.; 36 Urologic Oncology Program & Uro-Oncology Research Laboratories, Samuel Oschin Comprehensive Cancer Institute, Cedars-Sinai Medical Center, Los Angeles, CA.; 37 Department of Chemistry, Department of Biochemistry and Molecular Biology, Institute for Biophysical Dynamics, University of Chicago, Chicago, IL.; 38 Howard Hughes Medical Institute, University of Chicago, Chicago, IL.; 39 Department of Pathology, Duke University, Durham, NC.; 40 Netherlands Cancer Institute, Oncode Institute, Amsterdam, the Netherlands.; 41 Arc Institute, Palo Alto, CA.; 42 McDonnell Genome Institute, Washington University, St. Louis, MO.; 43 Department of Internal Medicine, Washington University, St. Louis, MO.; 44 Department of Biomedical Engineering, Washington University, St. Louis, MO.; 45 Department of Human Genetics, Institute for Precision Health, UCLA, Los Angeles, CA.; 46 Jonsson Comprehensive Cancer Center, Departments of Human Genetics and Urology, University of California Los Angeles, Los Angeles, CA.; 47 Michael Smith Genome Sciences Centre, BC Cancer, Vancouver, BC, Canada.; 48 Department of Epidemiology and Biostatistics, University of California, San Francisco, San Francisco, CA.

## Abstract

Analysis of DNA methylation is a valuable tool to understand disease progression and is increasingly being used to create diagnostic and prognostic clinical biomarkers. While conversion of cytosine to 5-methylcytosine (5mC) commonly results in transcriptional repression, further conversion to 5-hydroxymethylcytosine (5hmC) is associated with transcriptional activation. Here we perform the first study integrating whole-genome 5hmC with DNA, 5mC, and transcriptome sequencing in clinical samples of benign, localized, and advanced prostate cancer. 5hmC is shown to mark activation of cancer drivers and downstream targets. Furthermore, 5hmC sequencing revealed profoundly altered cell states throughout the disease course, characterized by increased proliferation, oncogenic signaling, dedifferentiation, and lineage plasticity to neuroendocrine and gastrointestinal lineages. Finally, 5hmC sequencing of cell-free DNA from patients with metastatic disease proved useful as a prognostic biomarker able to identify an aggressive subtype of prostate cancer using the genes *TOP2A* and *EZH2*, previously only detectable by transcriptomic analysis of solid tumor biopsies. Overall, these findings reveal that 5hmC marks epigenomic activation in prostate cancer and identify hallmarks of prostate cancer progression with potential as biomarkers of aggressive disease.

**Significance:**

In prostate cancer, 5-hydroxymethylcytosine delineates oncogene activation and stage-specific cell states and can be analyzed in liquid biopsies to detect cancer phenotypes.

*See related article by Wu and Attard, p. 3880*

## Introduction

DNA methylation is a critical driver of cancer phenotypes. The addition of a methyl group at the 5-carbon position of cytosine (5-methylcytosine, 5mC) is associated with closed chromatin and repressed gene expression (1). As part of the process to reverse methylation, 5mC can be oxidized to 5-hydroxymethylcytosine (5hmC) by the ten-eleven translocated (TET) family of enzymes. The number of 5hmC modifications represents only a fraction of the total methylation modifications but unlike 5mC, 5hmC is enriched at transcriptionally active regions, such as gene bodies and the borders of promoters and enhancers (1–4). 5hmC modifications are associated with active expression for many, but not all, genes and may mark dynamically activated transcription rather than constitutively expressed house-keeping genes (5). The location of 5hmC modifications is highly tissue specific, and a coordinated shift in 5hmC patterns occurs throughout tissue differentiation. Specifically, 5hmC is reported to mark chromatin in a poised state at developmentally regulated genes (6), and 5hmC is more frequently found in genes that drive tissue differentiation and among tissue-specific transcription factors (7, 8). 5hmC may also be important in maintaining a pluripotent cell state and to mark lineage commitment (7, 9–12). Both a global loss of 5hmC and local increases of 5hmC in genes and enhancers have been reported in cancer transformation and progression (13–19). The analysis of 5hmC modifications may thus provide an opportunity to assess epigenetic activation throughout cancer progression.

Prostate cancer is one of the most common cancers world-wide, and metastatic castration-resistant prostate cancer (mCRPC) is the second leading cause of cancer mortality among men. While a number of studies have identified genomic drivers of mCRPC, recent studies suggest that epigenomic alterations play an equally important role in prostate cancer progression (20–24). However, the global and specific 5hmC changes that occur during prostate cancer initiation and progression are still poorly understood. We therefore investigated the 5hmC landscape in prostate cancer by profiling 145 tumors (52 localized and 93 mCRPC samples). We integrated these data with whole-genome sequencing (WGS), RNA sequencing (RNA-seq), and whole-genome bisulfite sequencing (WGBS) and linked the results to 5hmC profiling in cell-free DNA from 79 prostate cancer patients. Our findings reveal that 5hmC comprehensively marks epigenomic activation in prostate cancer and identifies hallmarks of prostate cancer progression with potential as a noninvasive liquid biomarker of aggressive disease.

## Materials and Methods

### Patients and samples

#### Samples from metastatic castration-resistant prostate cancer

Fresh-frozen metastatic castration-resistant tissue from metastatic sites and normal adjacent tissue biopsy samples were collected through a multi-institutional image-guided prospective biopsy trial (NCT02432001) and DNA was extracted as previously described (24, 25). Plasma sample collection and cell-free DNA (cfDNA) extraction of matched cfDNA samples were performed as previously described (26). Plasma samples from men with mCRPC before first line androgen signaling inhibitor therapy were collected and DNA extraction was performed as previously described (27).

#### Samples from localized prostate cancer

Localized prostate cancer samples were collected as part of the CPC-GENE cohort (Canadian Prostate Cancer Genome Network). Patient selection, sample collection and processing procedures were performed as previously described (22).

#### Samples from benign prostate

Raw 5hmC-seq data from benign prostate samples were obtained from NCBI Gene Expression Omnibus (GEO) with accession number GSE144530.

### 5-Hydroxymethylcytosine sequencing

Sequencing library preparation and 5hmC enrichment was performed as described previously (28). In brief, cfDNA was normalized to 10 ng total input, and 10–25 ng for tissue for each assay and ligated to sequencing adapters. The adapter ligated library was partitioned 80:20 to enable 5hmC enrichment and whole-genome sequencing to be performed on each partition. 5hmC bases were biotinylated via two-step chemistry and subsequently enriched by binding to Dynabeads M270 Streptavidin (Thermo Fisher Scientific). All libraries were quantified by Bioanalyzer dsDNA High Sensitivity assay (Agilent Technologies Inc) and Qubit dsDNA High Sensitivity Assay (Thermo Fisher Scientific) and normalized in preparation for sequencing.

Sequencing of 5hmC-enriched libraries as well as input control (equivalent to low-pass WGS) was done on an Illumina NextSeq550 with 75bp paired-end reads using version 2 reagent chemistry according to the manufacturer's instructions (Illumina). Twenty-four libraries were sequenced per flow cell to yield approximately 20 million paired-end reads.

### Data processing of 5hmC-seq

Demultiplexing was performed using the Illumina BaseSpace Sequence Hub to generate sample-specific FASTQ output, and FASTQ files from different lanes were merged per sample. Reads were aligned to GRCh38/hg38 (Illumina iGenomes GRCh38Decoy, containing hsd1 decoys but not _ALT sequences) using bwa-mem (version 0.7.15-r1140 and 0.7.17-r1188). Aligned BAM files were marked for duplicates using GATK Picard (version 2.23.8) and mapping quality was assessed with Qualimap (version 2.2.1). Aligned BAM files were further filtered for high-quality reads by removing duplicate reads and keeping only properly mapped and paired reads with MAPQ >30 (-f 0×003 -F 0xf0c -q 30). Orphan reads created by MAPQ filtering were removed. Filtered, sorted, and indexed BAM files were used for downstream analyses.

Peak calling was performed using MACS2 (version 2.2.6; ref. 29) with 5hmC-enriched and input control samples and *P* value cut-off was set to 0.00001 in paired-end mode used with default settings. Peaks in blacklist regions from ENCODE (ENCFF419RSJ) and not on chromosome 1–22, X and Y were removed. Peaks were annotated to genomic regions and closest gene using the ChIPseeker R package (30), using promoter definition of -2000/+500bp from transcription start site (TSS) and the Gencode v28 transcript model annotation. Annotated peak frequencies in genomic regions were averaged per sample type for visualization.

5hmC gene body counts were extracted for Gencode v28 genes using featureCounts from the Rsubread package (31). Counts were calculated for the entire gene body from gene start to gene end allowing multioverlap. Gene body counts were further standardized for sequencing depth and gene length to transcripts per million (TPM).

5hmC enrichment analysis over genomic regions was performed using NGSplot (v. 2.61; ref. 32) with both 5hmC-enriched sequencing and input control on a per sample basis and then averaged per sample type.

### Previously published data

RNA-seq data was aligned with STAR and quantified at the gene level for Gencode v28 transcripts as previously described (24). WGBS and WGS data were acquired and processed as previously described (24, 25, 33). Copy-number calls were extracted as average copy number over the Gencode v28 gene regions from calls made by copycat. Promoter methylation was calculated as the average CpG methylation in a region -2000/+500bp from gene start.

Raw sequence chromatin immunoprecipitation sequencing (ChIP-seq) data for *AR, FOXA1, HOXB13*, H3K27ac, H3K4me3, H3K4me2 and H3K27me3 was downloaded from the Sequence Read Archive (SRA; SRP194063; ref. 21). Reads with base quality score > 30 across all bases were aligned using bwa-mem (0.7.17) to build GRCh38/hg38 (Illumina iGenomes). The aligned reads were deduplicated and peaks were called using MACS2 (v.2.2.5), with a false discovery rate (FDR; *q* value) threshold of 0.01. Peaks in genomic blacklisted regions defined by ENCODE (ENCFF356LFX) were excluded and only peaks that were enriched at least ten-fold over background were kept for further analysis. Qualified peaks were merged using consensusSeekeR (v.1.16) in which at least two samples had one peak in the same region (34). Only samples with more than two epigenetic marks available were included in this analysis.

*ERG* ChIP-seq data was downloaded from GEO (GSE14097; ref. 35).

### Data analysis

All downstream analyses were performed in R 3.6.3, using RStudio.

Differential 5hmC analysis was performed using raw gene body counts and the DESeq2 R package (36), or the diffbind R package for peak regions (37). For analysis of adenocarcinoma versus treatment-emergent small cell neuroendocrine prostate cancer (t-SCNC), metastatic site was included as covariable.

Gene set enrichment analyses (GSEA) were performed using the pre-ranked method implemented in the fgsea R package using the fgseaMultilevel function (38), and gene sets were retrieved from the molecular signatures database (MSigDB; ref. 39) using the msigdbr interface (version 7.2). Unless noted otherwise, GSEA was run using the Cancer Hallmark Pathways, the Wikipathways, the neuroendocrine prostate cancer (NEPC) gene sets (40), the luminal/basal gene sets (41), and the PCa-GI gene set (42).

For gene expression modeling, RNA-seq gene expression data was log_2_(TPM+1) transformed, scaled and then modeled as a linear model of 5hmC-seq gene body counts [log_2_(TPM+1) transformed and scaled], promoter methylation (average CpG methylation), copy number, and number of predicted activating or inactivation single nucleotide variants (SNV) and structural variants (SV). Genes with missing data or without variable gene expression or 5hmC levels were set to NA.

Hierarchical clustering was performed using the top 10% varying log_2_(TPM+1) transformed 5hmC gene body counts from protein coding genes, median centred and scaled per gene, and clustered using Euclidean distance metric and ward.D2 linkage. Principal component analysis was performed on log_2_(TPM+1) transformed, median centered and scaled 5hmC gene body counts for the 10% or 20% most variable protein-coding genes.

Transcription factor binding analysis was performed using HOMER (version 4.11) findMotifsGenome.pl (43), with default settings except ‘-size given’ to search in the entire peaks, and ‘-bg consensus_peaks.bed’ to use all consensus peaks as background. Significant upregulated peaks in the T2E-pos samples were filtered by FDR < 0.01 and fold change > 1.0 for use in the motif search.

Mapping of peak enrichment to genomic regions and biological pathways was performed using the GREAT tool (version 4.0.4), with enriched peaks (FDR < 0.001 and fold change > 1.5) as input and the entire genome as background (44).

A 5hmC classifier for tumor cell content was trained by first selecting genes with strongest correlation between 5hmC gene body counts and tumor cell content in tissue (*P* ≤ 0.05 and Bonferroni adjusted *q* ≤ 0.00001), resulting in 877 genes. These genes were further used to develop a linear model with elastic net regularization using the glmnet and caret R packages.

5hmC Tissue Map scores were generated as previously described (7).

### Statistical analysis

Correlation was calculated using Spearman correlation if not otherwise noted. Differences between groups were tested with the Wilcoxon rank-sum test. FDR was calculated using the Benjamini–Hochberg method when applicable.

Survival analyses were performed using the survival R package, with visualization through the survminer package. Survival probability was modeled using the Kaplan–Meier method, with endpoint time from first-line androgen signaling inhibitor for mCRPC to death of any cause. Hazard ratios were modeled using the Cox proportional hazards model, and differences between groups were tested using the Wald test. Multivariable Cox regression was performed including age at mCRPC diagnosis, PSA at first-line ARSI, Hb at first-line ARSI, type of ARSI (enzalutamide or abiraterone), docetaxel for metastatic hormone-sensitive prostate cancer, time to CRPC from start of androgen deprivation therapy (ADT), presence of visceral metastases and ct-fraction (when applicable) as covariables.

All hypothesis testing was done with two-sided tests, when applicable.

### Targeted sequencing of cell-free DNA

Somatic cfDNA variants (SNVs and indels) required ≥10 unique sequencing reads and a variant allele fraction (VAF) of ≥1%. Base substitutions required a minimum average read mapping quality of 10. We additionally required the VAF of putative cfDNA variants to be ≥20× higher than the average same-position background error rate calculated from a set of leukocyte control samples, and ≥3× higher than the paired germline DNA (gDNA) allele frequency. The paired leukocyte sample must have had at least 20× raw sequencing depth at that position. All variants were manually inspected in Integrative Genomics Viewer, and protein-level consequences were predicted using ANNOVAR (45). We removed putative variants with features suggestive of sequencing artifacts, including an imbalance in forward/reverse read representation, 5′ or 3′ read-end clustering, and variants in genomic regions of low complexity. Copy number alterations were called as previously described. Structural variants were called using a split-read approach as previously described (https://github.com/annalam/breakfast; ref. 46).

Germline variants required a minimum of 8 supporting unique reads and a minimum VAF of 10%. We discarded germline variants with a population frequency ≥0.5% in KAVIAR or ExAC databases (47, 48). Only putatively deleterious germline variants (defined as frameshift insertions/deletions, splice site mutations, or stopgain mutations) in select DNA damage repair genes were considered.

Circulating tumor DNA (ctDNA) content was estimated as previously described (46). Briefly, we used each sample's highest VAF somatic mutation—excluding those on amplified genes (log-ratio >0.2) or allosomes—as a surrogate for total ctDNA content. Because VAF is elevated in cases of concomitant loss of heterozygosity (LOH), we conservatively assumed associated LOH in all cases (even when the precise copy number state was not resolvable due to low ctDNA purity). In this circumstance, ctDNA fraction and highest mutation VAF were related by the formula: ctDNA% = 2/(1 + VAF^–1^). In a minority of samples with clear copy-number evidence of ctDNA content but no detected somatic mutations, we instead leveraged the heterozygous single nucleotide polymorphism (SNP) allele frequencies in genes with apparent heterozygous loss.

In this study, we reported the somatic or germline mutation status of the following prostate-cancer driver genes: *AKT1, APC, AR, ATM, BRCA1, BRCA2, CDK12, CDK12, CTNNB1, FOXA1, KMT2C, KMT2D, MSH2, MSH6, PIK3CA, PMS2, PTEN, RB1, SPOP, TP53,* or *ZFHX3*. For copy number alterations, we reported variants in *AR, ATM, BRCA1, BRCA2, CCND1, CDK12, CHD1, CLU, MSH2/6, MYC, NCOA2, NKX3-1, PTEN, RB1, TP53,* or *MLH1*.

### Ethical approval

Samples from mCRPC patients were part of an Institutional Review Board approved prospective biopsy cohort (NCT02432001) and human studies were approved and overseen by the UCSF Institutional Review Board. All individuals provided written informed consent to obtain fresh tumor biopsies and to perform comprehensive molecular profiling of tumor and germline samples. Profiling of additional cfDNA samples from men with mCRPC was approved by the University of British Columbia Clinical Research Ethics Board with certificate number H18-00944 and all patients provided written informed consent. Samples from localized prostate cancer were part of the CPC-GENE cohort (Canadian Prostate Cancer Genome Network). Written informed consent, following guidelines from local Research Ethics Board (REB) and the International Cancer Genome Consortium, was obtained at time of clinical follow-up. Previously collected tumor tissues were used according to University Health Network REB-approved study protocols (UHN 11-0024-CE). The study was performed in accordance with the Declaration of Helsinki.

### Code availability

Custom code used in the manuscript is available at https://github.com/DavidQuigley.

### Data availability

5hmC-sequencing and targeted cfDNA sequencing data created during this study are available at the European Genome-Phenome Archive (EGA) with study number EGAS00001004942. Benign prostate 5hmC-sequencing data is available at GEO (GSE144530). Whole-genome sequencing and RNA sequencing of mCRPC tissue samples is available at dbGaP with study accession phs001648.v2.p1. Whole-genome bisulfite sequencing data is available at SRA with Bioproject number PRJNA479544. ChIP-seq data used is available at GEO (GSE130408, GSE14097).

## Results

### 5hmC-seq identifies prostate cancer–specific active transcription beyond total methylation and DNA structural variants

To investigate the global patterns of 5hmC in advanced prostate cancer, we profiled tissue samples from 93 men with mCRPC using selective labeling of 5hmC modifications and pulldown coupled with sequencing (5hmC-seq; ref. 28), and performed an integrative analysis using WGS, RNA-seq, and WGBS data from the same samples (Supplementary Fig. S1; Supplementary Tables S1 and S2). The mCRPC tissue samples are part of a well characterized multi-institutional prospective biopsy cohort (NCT02432001; refs. 24, 25).

**Figure 1.**
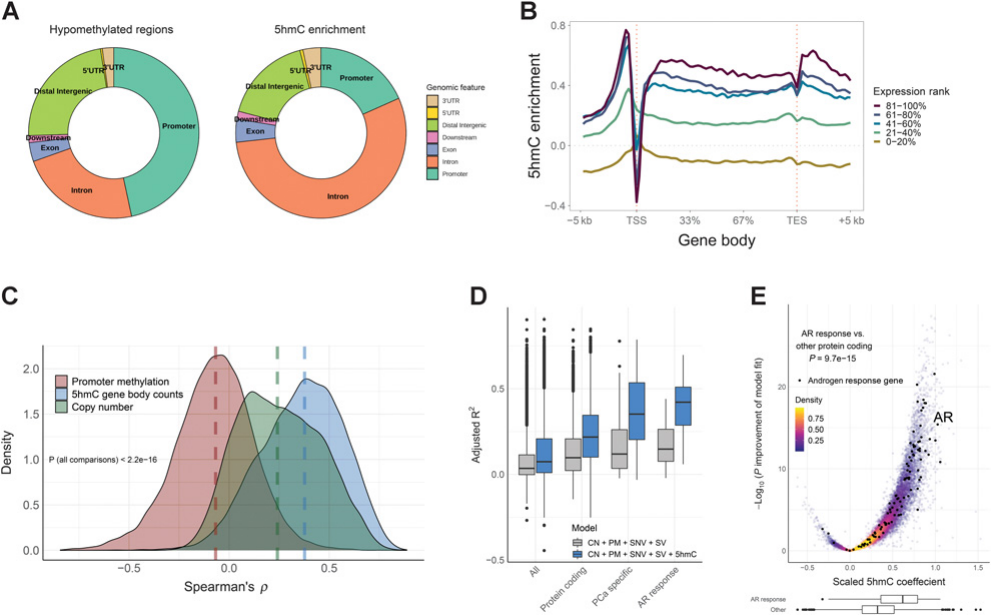
5hmC levels are enriched in gene bodies and are independently associated with gene expression in mCRPC. **A,** Location of hypomethylated regions defined by whole-genome bisulfite sequencing and location of 5hmC enrichment (peaks called by MACS2). Regions were mapped to Gencode v.28 transcripts per sample and the frequencies averaged across 93 mCRPC samples. **B,** 5hmC enrichment in and around gene bodies for different gene expression levels. Protein coding genes were assigned to expression quintile per sample and log_2_ 5hmC enrichment over input control (similar to low-pass WGS without 5hmC enrichment) was calculated using the NGSplot tool (32). 5hmC enrichment was then averaged across 93 mCRPC samples. **C,** Correlation between promoter methylation (average CpG methylation by whole-genome bisulfite sequencing), gene copy number, and 5hmC gene body counts, and gene expression, respectively, for protein coding genes across the 93 mCRPC samples. Genes with missing data or with no RNA-seq counts were excluded. Dashed lines, median correlation per data type. **D,** Gene expression was modeled for each gene by promoter methylation (PM), copy number (CN), SNVs, SVs, and 5hmC gene body counts (5hmC). Gene expression and 5hmC gene body counts were scaled (transformed to Z-score) to give comparable coefficients. Gray boxes, adjusted R-square of the model without 5hmC; blue boxes, adjusted R-square of the model including 5hmC. Analysis was done for 93 mCRPC samples. Boxplot shows median with hinges at 25th and 75th percentiles and whiskers at largest/smallest value within 1.5 × interquartile range. **E,** The adjusted 5hmC coefficients for individual genes modeled as in **D**. Genes in the Hallmark androgen response pathway are labeled black. *P* value was calculated by two-sided Wilcoxon rank-sum test for difference in scaled 5hmC coefficients between genes in the androgen response pathway including *AR* (*N* = 98) and all other protein coding genes (*N* = 18,434). Boxplots show distribution of AR response genes vs. other protein coding genes.

First, we explored the genome-wide 5hmC distribution in mCRPC and found that 5hmC peaks were located primarily in gene bodies and promoters (Fig. 1A). Since hypomethylated regions (HMR) measured by WGBS may indicate active gene regulatory regions (24), we compared the distribution of HMRs with 5hmC peaks and found that 5hmC peaks were located in gene bodies (exons or introns, excluding promoter overlap) more frequently than HMRs (mean across samples 59% vs. 27%, Wilcoxon rank-sum test *P* = 5.1 × 10^–32^, Fig. 1A), while HMRs were more frequently found in promoter regions (47% vs. 18%, Wilcoxon rank-sum test *P =* 5.1 × 10^–32^, Fig. 1A). Protein-coding genes with higher levels of 5hmC enrichment between the transcription start and end sites had higher levels of gene transcription measured by RNA-seq (Fig. 1B). 5hmC was also enriched at borders of CpG islands, promoters, enhancers, and HMRs close to actively transcribed genes (Supplementary Fig. S2).

Given that 5hmC enrichment was primarily found in gene bodies, we next sought to quantify the association between 5hmC gene body levels and gene expression. We calculated the correlation between gene expression and 5hmC gene body levels, copy number and promoter methylation by WGBS, respectively. 5hmC gene body levels had the strongest correlation with gene expression (median Spearman *ρ* for 5hmC 0.38, copy number 0.24 and methylation −0.07, respectively, *P* < 2.2 × 10^–16^; Fig. 1C). To identify biological pathways where 5hmC was most informative of RNA abundance, we performed a GSEA of genes ranked by strength of correlation between 5hmC and gene expression among the MSigDB Cancer Hallmark gene sets (39). The most statistically enriched pathway was androgen response, indicating that assessing 5hmC levels is informative of disease-specific genes, as dysregulation of androgen receptor (AR) signaling is a major mechanism of achieving castration-resistance in prostate cancer (Supplementary Fig. S3A; ref. 49).

We next tested if 5hmC-seq provides independent information to WGBS and WGS for predicting gene expression by integrating 5hmC gene body levels with promoter methylation, gene copy number, SNVs, and SVs. The addition of 5hmC data significantly improved the prediction of RNA abundance for most protein coding genes (FDR < 0.05 for 10,637/18,532 genes with complete data) and increased the median model fit (adjusted R^2^, percent explained after adjusting for number of predictors) by 0.12 when adding 5hmC (0.22 vs. 0.10, *P* < 2.2 × 10^–16^), which is similar to a previous report of 18% increase in prediction when combining 5hmC data and 5mC data in human benign tissues (Fig. 1D; ref. 50). In line with 5hmC modifications being most informative for disease relevant pathways, the improvement of fit when adding 5hmC data was considerably higher for genes overexpressed at the RNA level in prostate cancer (0.35 vs. 0.12, *P* = 1.1 × 10^–17^; ref. 24) and for genes in the androgen response pathway (0.42 vs. 0.15, *P* = 1.5 × 10^–18^; Fig. 1D), as well as several other Cancer Hallmark gene sets (Supplementary Fig. S3B). The scaled model coefficient, as a measurement of additional information provided by 5hmC, was also higher in the androgen response genes than other protein coding genes (*P* = 9.7 × 10^–15^; Fig. 1E).

Taken together, these analyses demonstrated that 5hmC levels are associated with transcriptional activity and provide orthogonal data to WGS and WGBS and is particularly informative for genes relevant in prostate cancer.

### 5hmC identifies molecular hallmarks of prostate cancer progression

**Figure 2.**
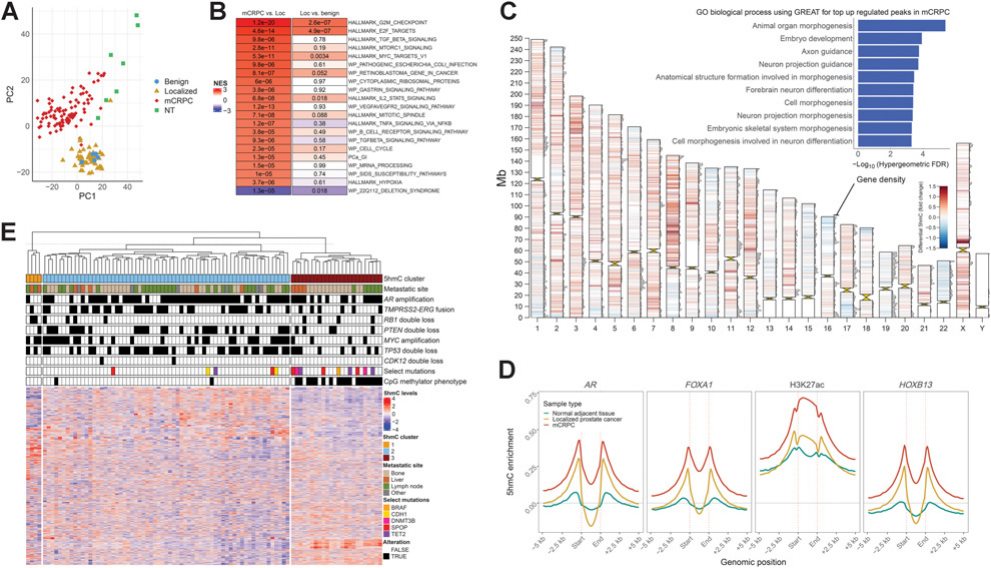
5hmC patterns change at different states and subgroups of prostate cancer. **A,** Unsupervised visualization of global 5hmC patterns for different prostate cancer states using principal component analysis for gene body counts of the top 10% variable protein coding genes. Benign, benign prostate tissue; Localized, localized prostate cancer; NT, normal adjacent tissue (to mCRPC biopsy). **B,** Differential 5hmC gene body analysis between mCRCP (*N* = 93) and localized prostate cancer (*N* = 52), and localized prostate cancer and benign prostate (*N* = 5), respectively. Genes were ranked by the DESeq2 statistic and further analyzed by GSEA. Color represents the normalized enrichment score (NES) and adjusted *P* values are shown for each pathway and state transition. Top significant pathways are shown. **C,** Differential 5hmC analysis for consensus peaks between localized prostate cancer and mCRPC. 5hmC peaks called by MACS2 for each sample were unified to a consensus set of peaks and used for differential analysis. Peaks with significant differences (FDR < 0.00001) are visualized per chromosome. Red, upregulation in mCRPC; blue, downregulation in mCRPC. Horizontal bars on chromosomes represent protein coding gene density. The most significant upregulated peaks (FDR < 0.001 and fold change > 1.5) in mCRPC were further analyzed by the GREAT tool for gene ontology biological processes, and the top 10 enriched biological processes by the hypergeometric test are shown as an inset (44). **D,** 5hmC enrichment at *AR*, *FOXA1, HOXB13*, and H3K27ac sites previously reported in mCRPC xenografts (21). 5hmC enrichment was calculated per sample and then averaged for localized prostate cancer (*N* = 52), normal adjacent tissue to mCRPC biopsies (*N* = 7), and mCRPC tissue samples (*N* = 93). **E,** Unsupervised hierarchical clustering of mCRPC tissue samples using 5hmC gene body counts of the top 10% most variable protein coding genes. Other, other metastatic soft tissue site. CMP, CpG methylator phenotype; Loc, localized prostate cancer.

To assess 5hmC changes associated with prostate cancer progression, we compared genome-wide 5hmC patterns in benign prostate (*N* = 5), localized castration-sensitive prostate cancer (*N* = 52), mCRPC (*N* = 93) and normal adjacent tissue (NT) to mCRPC biopsies (*N* = 7). Global 5hmC patterns were more similar between benign prostate and localized prostate cancer, while mCRPC had more distinct patterns likely reflecting both disease stage and disease site (Fig. 2A), and similar to previous observations of total methylation using WGBS data (24). A differential 5hmC gene body analysis between disease states identified genes in proliferative pathways to have a progressive increase in 5hmC levels during tumor progression, which was also observed for oncogenic pathways, such as *MYC* targets and *E2F* targets (Fig. 2B). 5hmC was increased in additional pathways in mCRPC such as *TGFβ* signaling and genes indicative of hypoxia, but not in localized prostate cancer, further supporting a more profound 5hmC dysregulation in mCRPC than in localized prostate cancer. Several known or putative driver genes were among the top hits, including *AR*, *EZH2*, *CDK1*, *TBX3*, and *HOXA13* (Supplementary Fig. S4). These results were not restricted to individual metastatic sites when stratifying the analysis for metastatic biopsy site, suggesting that 5hmC profiles associated with normal tissue types in the tumor microenvironment were not driving these results (Supplementary Fig. S5). Overall, these results using 5hmC-seq of DNA accurately capture the prostate cancer stage specific changes recently described on the transcriptional level (51).

Since 5hmC marked active regulatory regions in addition to gene bodies (Supplementary Fig. S2), we next interrogated tumor state–specific genome-wide 5hmC differences. Regions of 5hmC enrichment in mCRPC compared with localized castration-sensitive prostate cancer were distributed throughout the genome and were enriched for regulatory regions and genes driving developmental programs (Fig. 2C). These findings are in line with previous reports that epigenetic reprogramming is associated with dedifferentiation through reactivation of developmental programs during prostate cancer progression (21). To further corroborate this observation, we evaluated 5hmC levels at binding sites of the key prostate cancer oncogenes *AR, FOXA1* and *HOXB13* and H3K27ac modifications (from mCRPC xenograft data), and observed that 5hmC was enriched at these loci in mCRPC samples (Fig. 2D), suggesting that 5hmC marks the reported cistrome reprogramming associated with activation of developmental programs (21).

We further sought to explore 5hmC differences between subsets of mCRPC. An unbiased hierarchical clustering of gene body 5hmC levels identified three major clusters of samples (Fig. 2E). Cluster 1 consisted of samples that were previously characterized as t-SCNC, an aggressive prostate cancer subtype low in androgen signaling and with neuroendocrine features (52). Cluster 2 and 3 were hypo- and hypermethylated as determined by WGBS, respectively; cluster 3 largely corresponded to the previously described hypermethylated CpG methylator phenotype (CMP; ref. 24), with 18/20 CMP samples in cluster 3 (Fisher exact test *P* = 6.9 × 10^–12^, Fig. 2E). Differential 5hmC analysis between t-SCNC and adenocarcinoma confirmed known transcriptional differences such as lower 5hmC levels in genes in the androgen response pathway (FDR = 1.4 × 10^–7^), while genes previously identified to be active in NEPC (40) were the most upregulated (FDR = 4.4 × 10^–6^), together with additional neuronal gene sets (Supplementary Fig. S6).

In summary, these results suggest that 5hmC-seq can nominate novel putative driver genes and identify the activation of programs driving dedifferentiation, on top of recapitulating known molecular hallmarks previously seen at the transcriptomic, genomic and methylation levels.

### 5hmC delineates lineage plasticity and transdifferentiation

**Figure 3.**
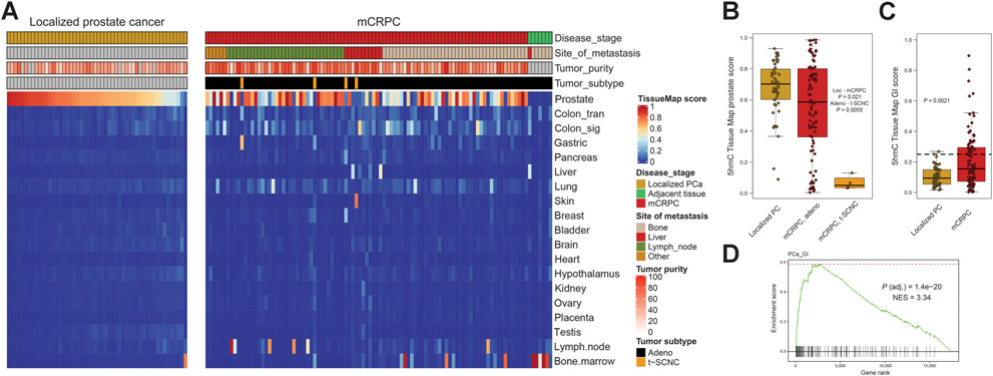
mCRPC lose prostate 5hmC marks and gain marks indicative of dedifferentiation and transdifferentiation. **A,** 5hmC tissue map scores were calculated for prostate cancer tissue samples predicting similarity to various tissues. Benign, benign prostate tissue; Localized, localized prostate cancer; NT, normal adjacent tissue (to mCRPC biopsy). Other, other metastatic soft tissue site. **B,** Box and whiskers plot for 5hmC tissue map prostate score for localized prostate cancer (*N* = 52), mCRPC adenocarcinoma (*N* = 89), and t-SCNC (*N* = 4). **C,** Box and whiskers plot for 5hmC tissue map combined gastrointestinal (GI) score (sum of the score in colon, gastric, liver and pancreatic tissue) for localized prostate cancer (*N* = 52) and mCRPC (*N* = 93). Horizontal line is drawn at a 5hmC GI score of 0.25, which classifies 4% of localized prostate cancer and 34% of mCRPC as having gained 5hmC GI patterns, similar to what has been previously reported at the gene expression level (42). **D,** GSEA for genes ranked by correlation between expression and the 5hmC GI score found the top pathway to be the previously described prostate cancer GI transcriptional signature. NES, normalized enrichment score. Boxplot shows median with hinges at 25th and 75th percentiles and whiskers at largest/smallest value within 1.5 × interquartile range.

Given that 5hmC has been suggested as a highly specific marker of lineage commitment during tissue development, and that prostate cancer can undergo lineage plasticity to escape AR-directed therapy (53), we next sought to evaluate 5hmC tissue specific markers in prostate cancer. Tissue-specific 5hmC profiles were recently characterized in a variety of normal tissues, leading to development of a method named the 5hmC tissue map that uses these profiles to predict tissue lineage based on 5hmC data (7). We applied this 5hmC Tissue Map and found that localized prostate cancer samples were largely classified as prostate tissue, which is consistent with the relatively similar global 5hmC patterns observed between benign prostate and localized disease (Fig. 3A). Normal tissue samples adjacent to metastatic biopsies were predicted to match that site's tissue of origin (i.e., bone marrow or liver), as expected (Fig. 3A). mCRPC samples retained varying levels of prostate-specific marks, and while mCRPC samples with low tumor fraction had a higher score of biopsy tissue of origin, loss of prostate-specific 5hmC marks was also observed for samples with high tumor fraction (localized prostate cancer vs. mCRPC *P* = 0.021, Fig. 3A and B). All four t-SCNC samples in this cohort had low 5hmC prostate scores (mCRPC adeno vs. mCRPC t-SCNC *P* = 0.0055, Fig. 3B). An additional one third of the mCRPC samples had a low prostate score and gain of 5hmC scores from gastrointestinal (GI) tissues (localized prostate cancer vs. mCRPC *P* = 0.0021, Fig. 3C). Aberrant activation of a GI gene expression circuit (PCa-GI transcriptional signature) has been previously suggested as a mechanism that enables cells to escape androgen signaling dependence and become resistant to treatments targeting AR (42). Expression of these PCa-GI genes were strongly correlated with the 5hmC GI patterns (enrichment for stronger correlation NES = 3.34, adjusted *P* = 1.4 × 10^–20^, Fig. 3D). Taken together, our data support the use of 5hmC patterns to accurately track prostate cancer lineage plasticity through loss of prostate specific 5hmC patterns.

### 5hmC marks the activation of specific cancer driver genes and transcriptional programs

**Figure 4.**
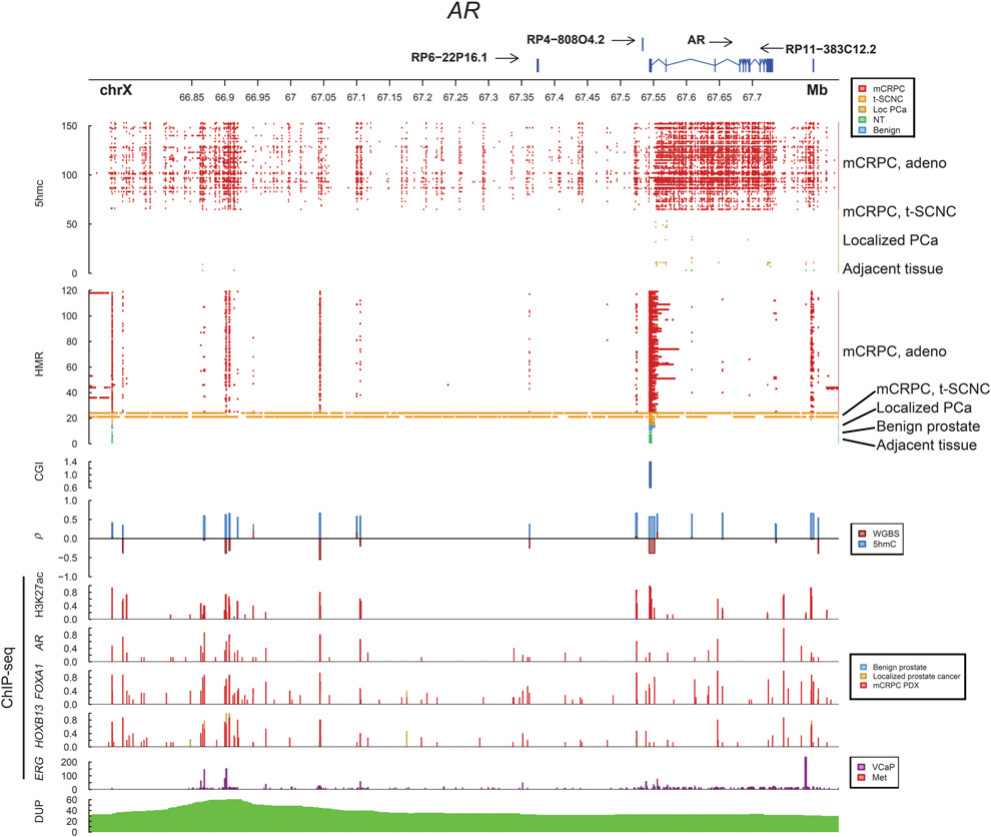
5hmC marks activity of the androgen receptor locus. Integration of multiple layers of data for the *AR* locus. 5hmC represents peaks called by MACS2 for each sample. HMR, hypomethylated regions called by whole-genome bisulfite sequencing per sample. CGI, CpG islands; *ρ*, Spearman correlation between 5hmC peaks and gene expression, and methylation levels by whole-genome bisulfite sequencing, respectively. ChIP-seq from publicly available patient samples, patient-derived xenografts, and cell lines. DUP, number of mCRPC samples with tandem duplications; PDX, patient-derived xenograft. Benign, benign prostate tissue; Localized, localized prostate cancer; NT, normal adjacent tissue (to mCRPC biopsy); PCa, prostate cancer.

Since 5hmC marks genes and regulatory regions corresponding to transcriptional activity, we sought to further explore the detailed 5hmC patterns of key prostate cancer driver genes. The AR is the primary therapeutic target in prostate cancer; copy number increases affecting the *AR* gene body and distant enhancer produce upregulation of AR expression in mCRPC after tumors escape ADT (25). 5hmC marked the *AR* gene body in mCRPC, but not in localized prostate cancer, and 5hmC was absent from the *AR* gene body of t-SCNC tumors, corresponding to known *AR* activity levels in these subsets of tumors (Fig. 4). Further, 5hmC marked the known *AR* enhancers in mCRPC, including the putative novel enhancers identified by hypomethylation called by WGBS (24, 25), and 5hmC levels at these regions were positively correlated with expression of *AR*.

The pioneering factor FOXA1 modulates chromatin accessibility, directly interact with AR and shapes its signalling driving prostate cancer tumor growth. *FOXA1* activity can be altered either by aberrations to the gene itself or of the surrounding regulatory regions (54, 55). The *FOXA1* gene body was hypomethylated, while 5hmC marked the region upstream of the TSS both in localized prostate cancer and mCRPC (Supplementary Fig. S7). Several *FOXA1* downstream regulatory regions were hypomethylated and marked by 5hmC, potentially indicating activity. Notably, *FOXA1* is commonly mutated in localized prostate cancer (20), while *AR* is frequently altered in response to ADT, and there are more altered *AR* binding sites in mCRPC than altered *FOXA1* sites (21). Thus, the profoundly altered 5hmC patterns around *AR* in mCRPC vs. localized prostate cancer and relatively similar *FOXA1* patterns are consistent with their reported degree of dysregulation at different disease states.

**Figure 5.**
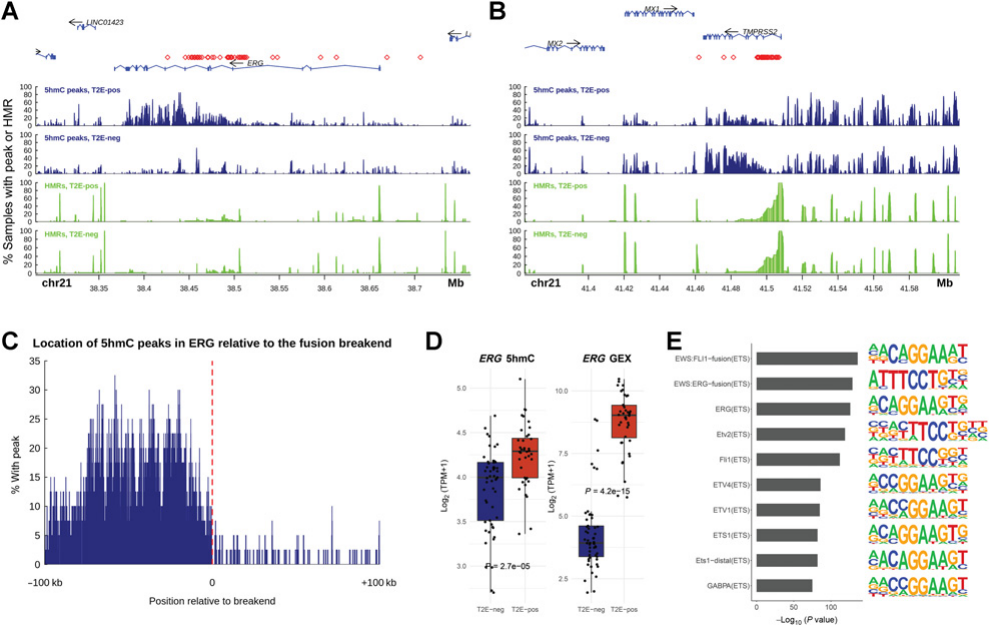
Activating *TMPRSS2-ERG* fusions and the downstream cistrome are marked by 5hmC. **A** and **B,** 5hmC locations and hypomethylation at the *ERG* and *TMPRSS2* loci. 5hmC levels are shown as frequency of samples, with a 5hmC peak called by MACS2 at each position and hypomethylation as frequency of samples with a HMR called from whole-genome bisulfite sequencing. Samples are split based on the presence of a *TMPRSS2-ERG* fusion (T2E). Red symbols mark the position of the 3′ and 5′ break-ends for each sample harboring a *TMPRSS2-ERG* fusion. **C,** Location of 5hmC peaks are shifted relative to the 3′ break-end in *TMPRSS2-ERG* fusion positive samples. **D,***ERG* 5hmC gene body levels and gene expression per fusion status (T2E-negative, *N* = 53; T2E-positive, *N* = 40). Two of the T2E-negative samples had a *SLC45A3-ERG* fusion. **E,** The top 10 enriched transcription factor binding motifs analyzed by HOMER for loci that have enriched 5hmC levels in *TMPRSS2-ERG* fusion–positive samples. GEX, gene expression; T2E-pos, samples harboring a *TMPRSS2-ERG* gene fusion; T2E-neg, samples not harboring a *TMPRSS2-ERG* gene fusion. Boxplot shows median with hinges at 25th and 75th percentiles and whiskers at largest/smallest value within 1.5 × interquartile range.

To further analyze how 5hmC marks different mechanisms of gene activation, we studied the activating *TMPRSS2-ERG* fusion (T2E), a somatic alteration present in ∼50% of prostate tumors (56). Through this fusion, the promoter of the AR-regulated gene *TMPRSS2* fuses with the ETS-family transcription factor *ERG*, or other ETS-family members, thus making *ERG* an *AR-*regulated gene. We found an increase of 5hmC modifications in *ERG* downstream of the fusion break end in T2E-positive samples (Fig. 5A–C), and *ERG* gene expression and *ERG* 5hmC levels were higher in fusion positive samples (Fig. 5D). When comparing global 5hmC patterns between T2E positive and negative samples using differential peak analysis and the HOMER motif analysis tool (43), we found regions with increased 5hmC in T2E-positive samples to be strongly enriched in ETS-family transcription factors binding motifs (Fig. 5E). A detailed analysis of the canonical *ERG* target gene *KCNS3* (57) revealed that both gene expression and 5hmC gene body levels were increased and that regions with significant 5hmC enrichment in and around *KCNS3* were predicted to harbor *ERG* binding motifs (Supplementary Fig. S8A and S8B). Collectively, these data suggest that 5hmC marks the activation of major driver genes in mCRPC, both the gene bodies and regulatory sites, as well as downstream target binding sites.

### Prostate cancer–specific 5hmC patterns are detectable in cfDNA

**Figure 6.**
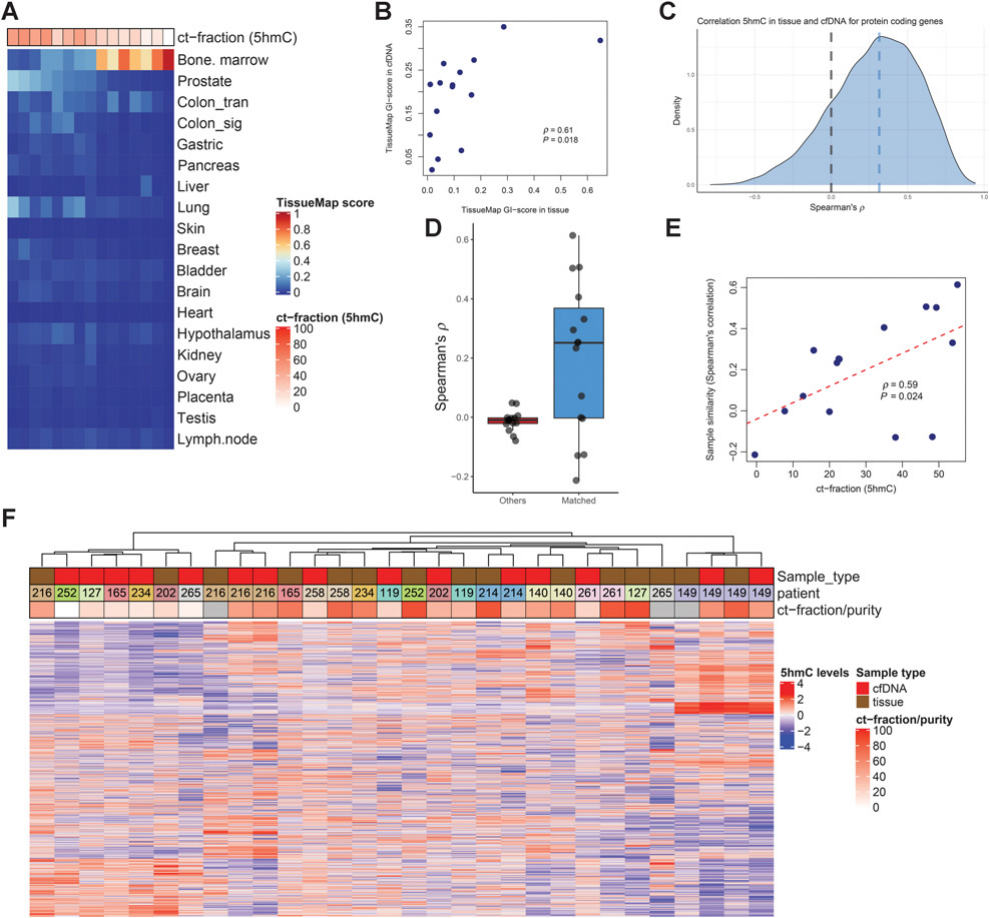
Concordance of 5hmC patterns in 15 matched tissue and cfDNA samples. **A,** Tissue map scores for 15 cfDNA samples with matched tissue 5hmC-sequencing available. Predicted ct-fraction was determined using a novel 5hmC-classifier. Patients DTB-149 and DTB-216 had two paired samples at two different time points available with 5hmC sequencing, but not other data modalities and were thus included in this paired analysis but not in integrative analyses. **B,** Scatterplot for tissue map 5hmC GI-score in tissue and cfDNA for the 15 matched pairs of samples. **C,** Spearman correlation between tissue 5hmC gene body counts and cfDNA gene body counts. Blue dashed line, median correlation. **D,** Box and whiskers plot showing correlation for 5hmC gene body counts in tissue and cfDNA for matched pairs (*N* = 15, blue box) and for average correlation to nonmatched pairs (*N* = 15, red box). **E,** Scatterplot for sample similarity of matched tissue and cfDNA samples (*N* = 15) measured by Spearman correlation for 5hmC gene body counts of protein coding genes and predicted ct-fraction by 5hmC levels. **F,** Hierarchical clustering of matched 5hmC gene body counts in tissue and cfDNA samples using the top 10% most variable genes. Scaling (z-scores) was performed separately for cfDNA and tissue-derived samples. ct-fraction/purity is estimated from 5hmC-sequencing (cfDNA samples) or from WGS (tissue). Boxplot shows median with hinges at 25th and 75th percentiles and whiskers at largest/smallest value within 1.5 × interquartile range.

Given the evidence that 5hmC levels of DNA are informative of transcriptional activity, activation of driver genes and programs, and dedifferentiation of mCRPC, we next investigated whether 5hmC levels in cfDNA were representative of the corresponding tumor tissue. We performed 5hmC-seq on 15 cfDNA samples for which we had a matching mCRPC biopsy profiled with 5hmC. Since cfDNA is a mixture of DNA originating from the tumor (circulating tumor DNA, ctDNA) and cfDNA from normal cells (mostly leukocytes), we first hypothesized that we could use 5hmC patterns to predict the circulating tumor fraction (ct-fraction) of cfDNA. The 5hmC-predicted ct-fraction was strongly correlated with 5hmC tissue map prostate score (*ρ* = 0.89, *P* < 2.2 × 10^–16^) and anticorrelated with 5hmC tissue map bone marrow score, as expected (*ρ* = −0.84, *P* = 8.1 × 10^–5^; Fig. 6A). We further observed GI patterns in cfDNA from men with mCRPC that paralleled those observed in tissue, and there was a positive correlation between GI patterns in tissue and cfDNA (*ρ* = 0.61, *P* = 0.018; Fig. 6B).

We next compared the correlation in gene body 5hmC levels between tissue and cfDNA for all protein coding genes and found a positive correlation for most genes (Fig. 6C). Samples with higher predicted ct-fraction in cfDNA had a stronger association between cfDNA and solid tumor 5hmC levels (*ρ* = 0.59, *P* = 0.024; Fig. 6D and E). Unbiased clustering of matched cfDNA and tissue samples demonstrated that cfDNA samples with higher predicted ct-fraction were more frequently clustered together with their matched solid tumor (Fig. 6F). Thus, these data indicate that 5hmC patterns in cfDNA are representative of the individual mCRPC tumors, and, as is the case with targeted sequencing and 5mC analysis of cfDNA (58, 59), the concordance is stronger in patients with higher ct-fraction.

### 5hmC is a potential liquid biomarker for mCRPC

**Figure 7.**
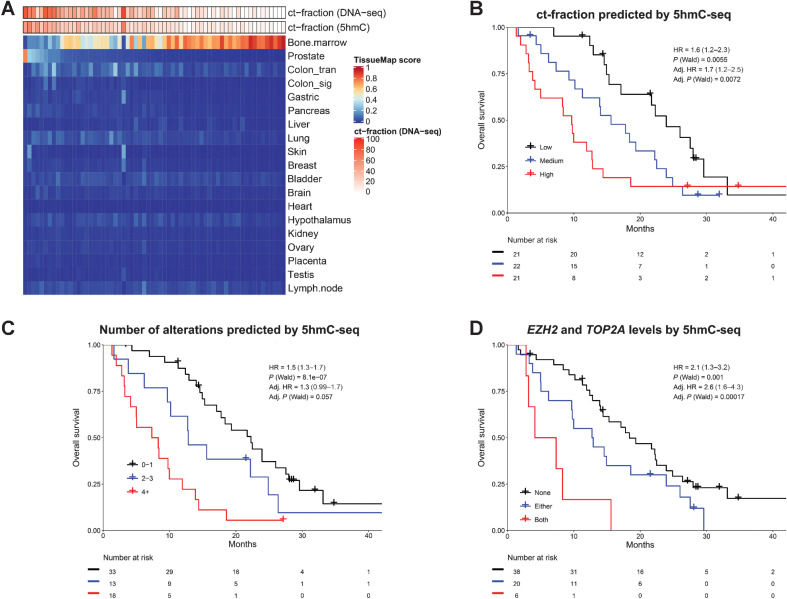
5hmC in cell-free DNA of patients with mCRPC. **A,** 5hmC tissue map scores for each of the 64 cfDNA samples taken before first-line androgen receptor signaling inhibitor (enzalutamide or abiraterone) for mCRPC. Circulating tumor fraction (ct-fraction) was estimated from a targeted cfDNA sequencing panel and by 5hmC-seq using a novel classifier based on gene body 5hmC counts. **B,** Overall survival for patients split by tertiles of 5hmC predicted ct-fraction. **C,** Overall survival based on the number of genomic events inferred by 5hmC gene body counts of the eight most commonly altered genes by targeted cfDNA sequencing. Oncogenes (*AR*, *MYC*, *NCOA2*) were considered gained if 5hmC gene body counts were in the upper quartile across samples, and tumor-suppressor genes (*RB1*, *PTEN*, *TP53*, *BRCA2*, *NKX3-1*) were considered lost if 5hmC gene body counts were in the lowest quartile. Kaplan-Meier curves are visualized for 0–1, 2–3 and >3 events, and HRs are calculated as mean for each additional event inferred. **D,** Overall survival based on 5hmC gene body levels of *TOP2A* and *EZH2*. Levels are split by quartiles across samples and survival is contrasted between samples being in the top quartile for none, either, or both *TOP2A* and *EZH2*, as previously described for tissue gene expression (61). *P* values were calculated by two-sided Wald test. Adjusted HRs are adjusted for ct-fraction, age at mCRPC diagnosis, PSA at first-line ARSI, Hb at first-line ARSI, type of ARSI (enzalutamide or abiraterone), docetaxel for metastatic hormone-sensitive prostate cancer, time to CRPC from start of ADT, and presence of visceral metastases.

To further evaluate 5hmC in cfDNA as a liquid biopsy biomarker in mCRPC, we profiled an additional cohort of 64 cfDNA samples from a series of clinically well-annotated patients before first-line androgen signaling inhibitor therapy (ARSI; abiraterone or enzalutamide) with both 5hmC-seq and a targeted cfDNA-panel (Supplementary Table S3). First, we evaluated the global 5hmC patterns by performing a principal component analysis and applying the 5hmC ct-fraction and tissue map classifiers to the cfDNA samples (Fig. 7A; Supplementary Fig. S9A and S9B). The first principal component was associated with ct-fraction, and the predicted ct-fraction from 5hmC was highly correlated with the estimated ct-fraction from targeted cfDNA-seq (*ρ* = 0.83, *P* = 1.1 × 10^–17^). 5hmC tissue map prostate score was correlated with ct-fraction (*ρ* = 0.84, *P* = 1.8 × 10^–18^), while bone marrow score was anti-correlated (*ρ* = −0.69, *P* = 3.7 × 10^–10^). These results highlight that 5hmC patterns are very specific for prostate cancer-derived DNA enabling the accurate quantification of ct-fraction, which is clinically informative as higher ct-fraction determined by 5hmC-seq was strongly prognostic for outcome [Fig. 7B, per tertile of ct-fraction, HR = 1.6 (1.2–2.3), *P* = 0.006], similar to ct-fraction estimated from targeted DNA-seq (Supplementary Fig. S9C). We also observed the gastrointestinal 5hmC patterns in cfDNA in this cohort (Fig. 7A). One sample (UBC1) had a high ct-fraction, but did not exhibit any prostate 5hmC patterns, and instead had higher scores of colon, gastric, skin, and breast tissues. This sample harbored multiple genomic alterations consistent with aggressive prostate cancer (determined by targeted cfDNA-seq in *AR*, *MYC*, *NCOA2*, *RB1*, *TP53*, *NKX3-1*, *BRCA2*, and *PTEN*; Supplementary Table S3), suggesting that dedifferentiation of aggressive mCRPC can be detected in cfDNA by 5hmC-seq.

The number of altered pathways in aggressive prostate cancer is prognostic (60); we therefore created a score in cfDNA using 5hmC-seq inferred gain or loss of the eight most commonly altered prostate cancer driver genes by targeted cfDNA-seq (*AR, MYC*, *NCOA2*, *RB1*, *TP53*, *BRCA2*, *PTEN*, *NKX3-1)*. This score was prognostic for overall survival [per additional genomic event, HR = 1.5 (1.3–1.7), *P* = 8.1 × 10^–7^, adjusted for ct-fraction and clinical covariables: HR = 1.3 (0.99–1.7), *P* = 0.057; Fig. 7C]. These results were concordant with those obtained by targeted cfDNA-seq (Supplementary Fig. S9D).

Finally, we explored whether 5hmC-seq can be used to infer activity of genes that are not commonly altered at the DNA level in prostate cancer, rendering them opaque to current cfDNA-seq methods. We have previously shown that the tissue gene expression levels of the genes *TOP2A* and *EZH2* can identify an aggressive subgroup of prostate cancer (61). *EZH2* was one of the top differentially 5hmC marked genes in mCRPC versus localized prostate cancer. Neither gene is frequently altered by copy number changes in mCRPC; *EZH2* had DNA alterations in 0.5% of 1465 samples, while *TOP2A* is not commonly included in prostate cancer targeted panels (62). Using 5hmC levels in cfDNA, the previously described gene expression classification of the combined genes was highly prognostic for overall survival after adjusting for ct-fraction and clinical variables [adjusted HR = 2.6 (1.6–4.3), *P* = 0.0002; Fig. 7D].

In summary, our data indicate that 5hmC-seq can be used to detect cancer specific 5hmC patterns in cfDNA to accurately estimate ct-fraction as well as to find specific gene activation of driver genes not altered at the DNA level, thus potentially adding to current analyses of cfDNA for advanced cancers.

## Discussion

In this study, we have characterized the genome-wide 5hmC landscape of prostate cancer at different disease states, comprehensively modeled transcriptional regulation by integrating 5hmC-seq with WGS, WGBS, and RNA-seq, and explored 5hmC-seq of cfDNA as a liquid biomarker for advanced prostate cancer. To our knowledge, this study represents the only integrated analysis of 5hmC with genome-wide and epigenome-wide sequencing approaches in clinical tumor samples, and the first study to comprehensively profile 5hmC marks in a large cohort of cancer metastases. We found that 5hmC is a marker of epigenomic activation throughout disease progression, identifying distinct oncogenic signaling pathways that define disease states as well as subgroups of advanced prostate cancer. 5hmC is enriched not only at driver gene bodies but also at specific downstream target genes and binding sites, further supporting 5hmC as a general marker of epigenetic activation and cancer cell reprogramming. Furthermore, 5hmC identifies dedifferentiation and lineage plasticity, which are critical mechanisms of therapy resistance. We also demonstrated that 5hmC analysis of cfDNA of mCRPC patients accurately detects prostate cancer specific 5hmC patterns that are prognostic. Finally, 5hmC-seq may be used to develop liquid biomarkers for genes not commonly altered at the DNA structural level.

Our data indicate that 5hmC can be used to assess the epigenomic state of the tumor by identifying transcriptionally activated genes and programs. These findings using 5hmC-seq are similar to the current understanding of prostate cancer progression at the transcriptional level (51), but critically do not require the isolation of RNA for gene expression profiling and has potential for immediate translational impact through the analysis of cfDNA. The role of 5hmC at these locations is still under investigation, and while 5hmC is enriched at regions undergoing active demethylation, 5hmC is also reported to be stable enough for a possible transcription-promoting function (63). Indeed, 5hmC is suggested to alter the binding affinity of DNA-binding proteins resulting in “functional demethylation” (64). In our analysis, 5hmC marked driver genes, genes overexpressed as a consequence of structural variants, downstream target genes and regulatory regions associated with driver genes. This supports the role of 5hmC as an epigenomic mark of oncogenic activation. Future mechanistic studies will be crucial to further understand the biological role of dynamic 5hmC changes over time, and if it may directly promote transcription.

While we found that localized prostate cancer retained prostate specific 5hmC patterns, advanced castration-resistant metastatic prostate cancer appeared more dysregulated with variable loss of prostate 5hmC patterns. These changes may be associated with both the progression from localized to metastatic disease, as well as the development of castration resistance. All t-SCNC mCRPC samples lost their prostate 5hmC patterns, but an additional one third of adenocarcinoma mCRPC samples had low prostate 5hmC patterns and gain of patterns associated with gastrointestinal tissue. While several epigenomic studies have been performed in prostate cancer, 5hmC appears to more robustly mark unique lineages such as this gastrointestinal subset. Moreover, 5hmC sequencing highlights developmental pathways shaping cancer progression and dedifferentiation that are not as readily seen with transcriptional profiling. This is consistent with studies of 5hmC in tissue differentiation that found that 5hmC marks key genes associated with both maintaining pluripotency and lineage commitment with 5hmC marking distinct programs at certain developmental timepoints (11). Prostate cancer transdifferentiation has been proposed as a mechanism by which tumors acquire treatment resistance to AR targeting agents by reducing dependence on androgen signaling. This phenomenon has been studied in the context of a switch to a small cell and/or neuroendocrine phenotype (53), but other mechanisms have been reported such as a lineage switch towards a gastrointestinal phenotype (42), and recent studies have confirmed the role of the epigenome in regulating dedifferentiation in prostate cancer (21, 65). Our data indicate that 5hmC analysis could further help delineate mCRPC de- and transdifferentiation and could potentially be used to track prostate cancer lineage plasticity in liquid biopsies.

In clinical practice, biopsies of prostate cancer metastases can be difficult to obtain, and liquid biomarkers are more readily accessible. While 5hmC-seq is concordant with common DNA alterations, it can also infer activity of additional genes. We studied the previously defined subgroup of tumors with high expression of *TOP2A* and *EZH2* (61), and we found that the classification in cfDNA by 5hmC-inferred activity levels of *TOP2A* and *EZH2* was highly prognostic. Given that neither *TOP2A* nor *EZH2* commonly harbor somatic changes in prostate cancer that can be detected by DNA sequencing, this highlights the potential of 5hmC-seq to add to current analyses of cfDNA in advanced cancers. Future studies will be important to establish lower bounds for the amount of circulating tumor DNA required to obtain consistent results from cfDNA analysis of 5hmC patterns. In total, our study establishes how 5hmC fits into the genomic, epigenomic, and transcriptomic context of prostate cancer progression and demonstrates the potential as a liquid biopsy platform for clinical biomarker development.

## Supplementary Material

Supplementary Figure

Supplementary Table

Supplementary Table

Supplementary Table
