# Supplementary material for: The 5-Hydroxymethylcytosine Landscape of Prostate Cancer
Source: Cancer Res. 2022 Oct 17;82(21):3888–902. doi: 10.1158/0008-5472.CAN-22-1123 (PMC9627125; doi:10.1158/0008-5472.CAN-22-1123)
Supplement: Supplementary Figure [file can-22-1123_supplementary_figures_suppsf1-sf9.pdf]

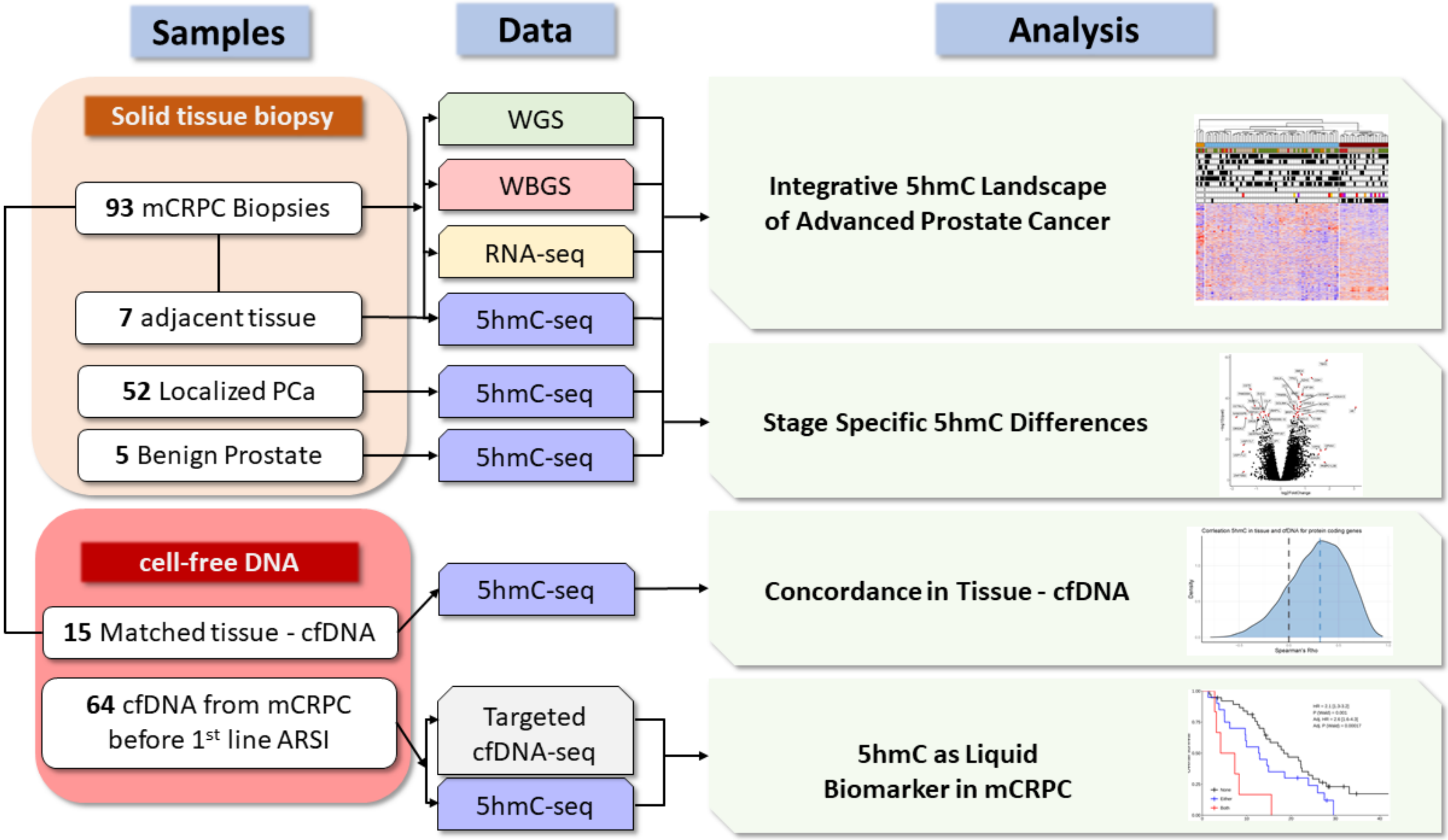

Figure S1. Study overview.
mCRPC; metastatic castration-resistant prostate cancer, PCa; prostate cancer, cfDNA; cell-free DNA, ARSI; androgen receptor signaling inhibitor, WGS; whole-genome sequencing, WGBS; whole-genome bisulfite sequencing.

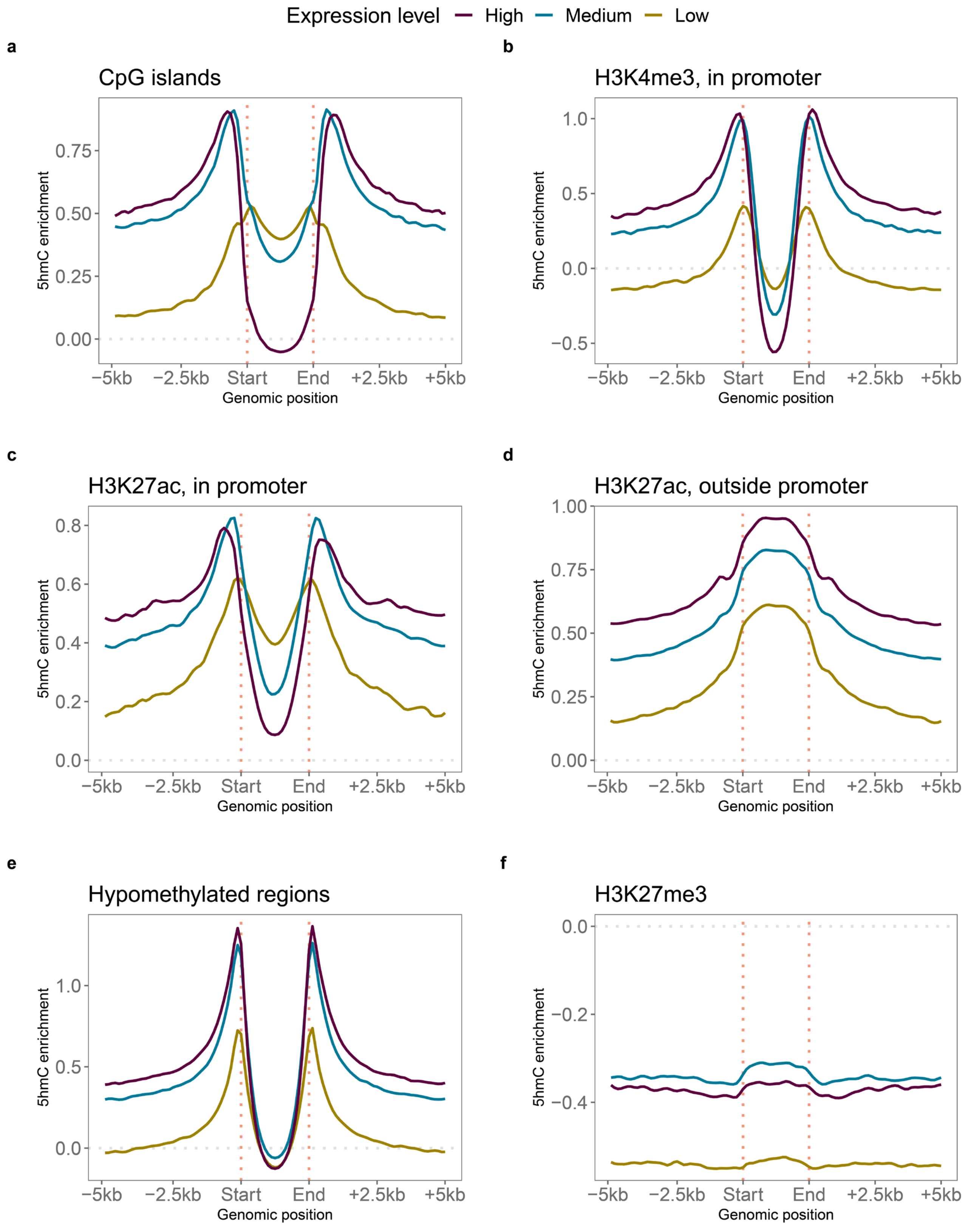

Figure S2. 5hmC enrichment at different genomic features.
The respective region types were mapped to the closest gene and genes were split by tertiles of expression levels per sample. Log2 5hmC enrichment over input control (similar to low-pass WGS without 5hmC enrichment) was calculated for each group of genes per sample and results were then averaged for the 93 mCRPC tissue samples. Regions analyzed were **a)** CpG islands, **b)** H3K4me3 marked regions overlapping promoters, **c)** H3K27ac marked regions overlapping promoters, **d)** H3K27ac marked regions not overlapping promoters (putative enhancers), **e)** Hypomethylated regions as defined by whole-genome bisulfite sequencing per sample, and **f)** H3K27me3 marks (considered representative of condensed chromatin). CpG islands annotation was from the UCSC Genome browser track and ChIP-seq data from publicly available data (1).

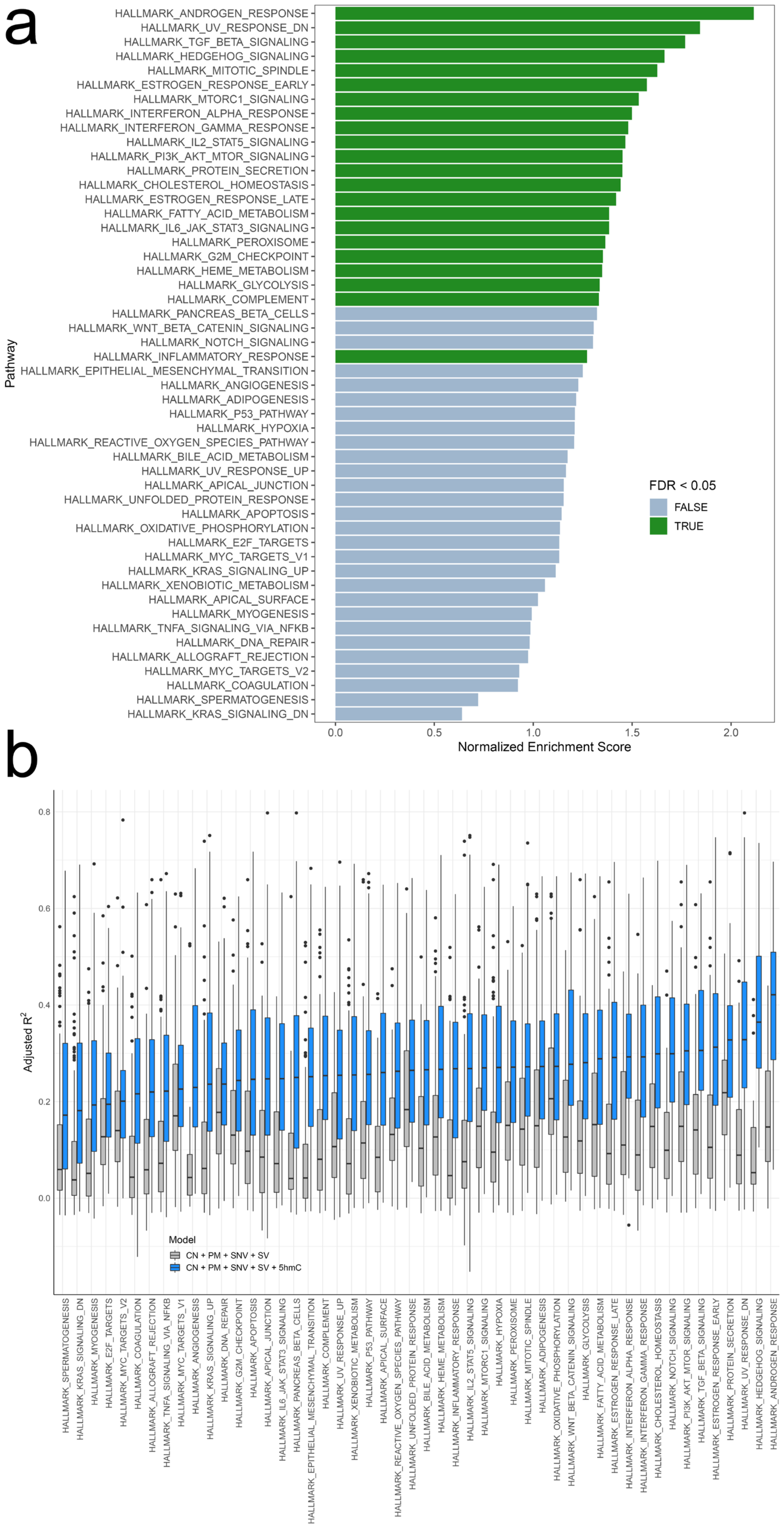

Figure S3. Gene expression and 5hmC levels for the Cancer Hallmark Pathways.
a) Gene set enrichment analysis for genes ranked by strength of 5hmC gene body count and gene expression correlation for the MSigDB Cancer Hallmark pathways. **b)** Gene expression was modeled for each gene by promoter methylation (PM), copy number (CN), single-nucleotide variants (SNV), structural variants (SV) and 5hmC gene body counts (5hmC). Gene expression and 5hmC gene body counts were scaled (transformed to Z-score) to give comparable coefficients. Grey boxes represent the adjusted R-square of the model without 5hmC while the blue boxes represent the adjusted R-square of the model including 5hmC. Analysis was done for 93 mCRPC samples. Boxplot shows median with hinges at 25th and 75th percentiles and whiskers at largest/smallest value within 1.5 * inter quartile range.

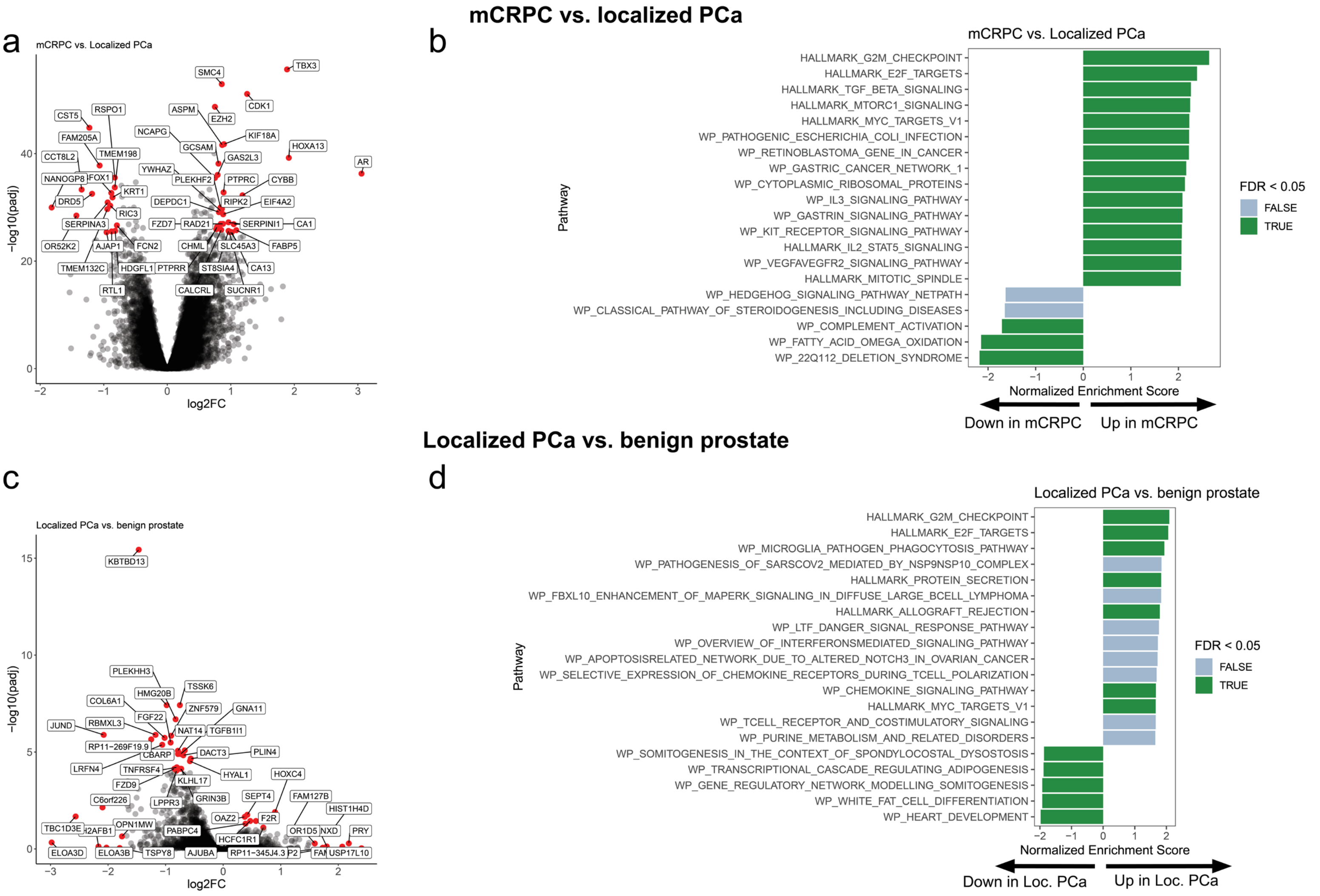

Figure S4. Differential 5hmC analysis between different states of disease.
a) Volcano plot of differential 5hmC gene body counts between mCRPC (N=93) and localized prostate cancer (N=52). Top differential genes are marked in red. **b)** Gene set enrichment analysis of differential 5hmC gene body counts between mCRPC and localized prostate cancer. The top 15 up regulated and bottom 5 down regulated pathways by normalized enrichment score are shown. **c)** Volcano plot of differential 5hmC gene body counts between localized prostate cancer and benign prostate (N=5). Top differential genes are marked in red. **d)** Gene set enrichment analysis of differential 5hmC gene body counts between localized prostate cancer and benign prostate. The top 15 up regulated and bottom 5 down regulated pathways by normalized enrichment score are shown.

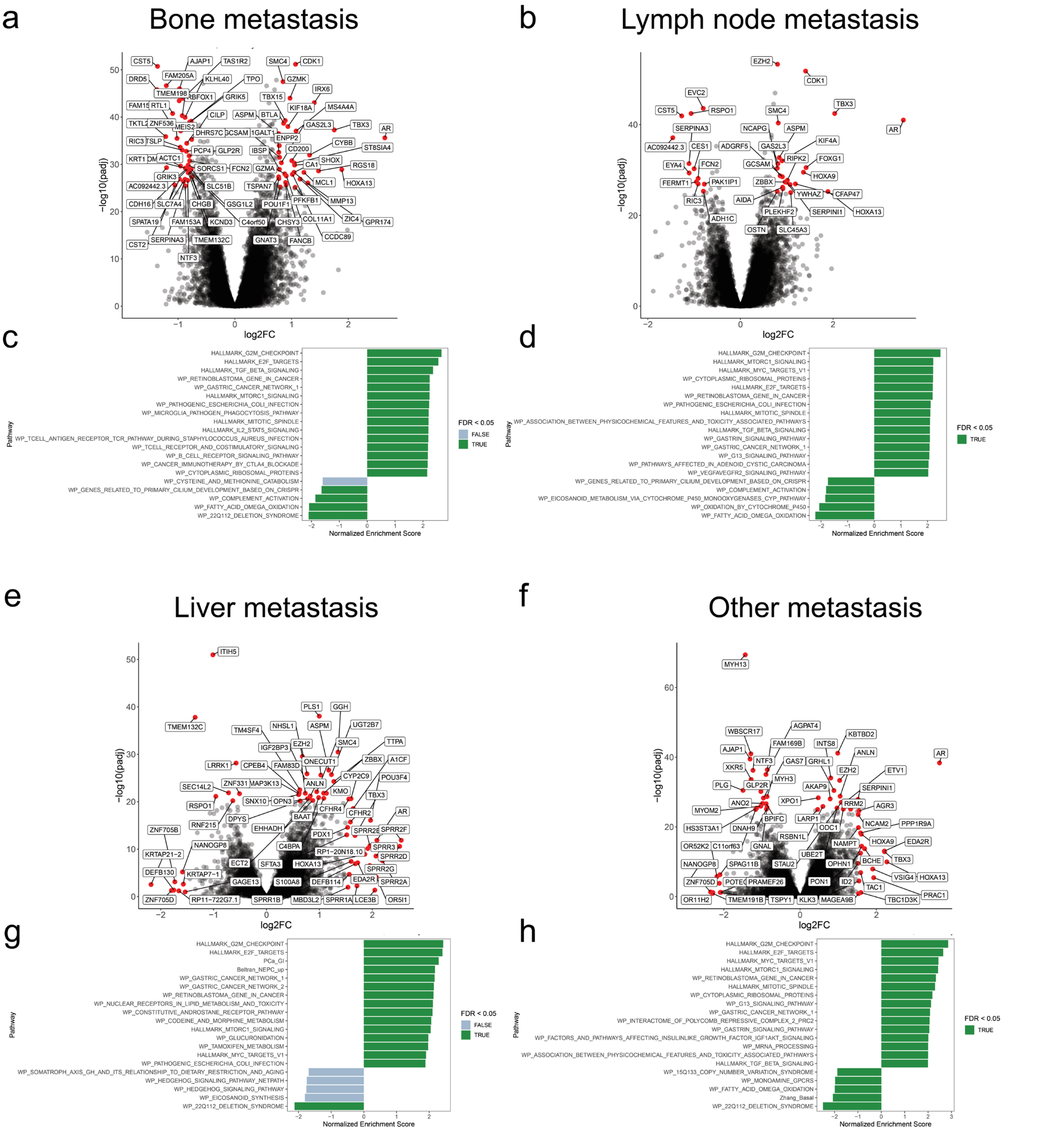

Figure S5. Differential 5hmC analysis between mCRPC and localized prostate cancer stratified for metastatic site.

a,b,e,f) Volcano plots of differential 5hmC gene body counts between mCRCP and localized prostate cancer (N=52), stratified for metastatic site (bone N=42, lymph node N=34, liver N=11, other N=6). Other means other metastatic soft tissue site. Top differential genes are marked in red. **c,d,g,h)** Gene set enrichment analysis of differential 5hmC gene body counts between mCRPC and localized prostate cancer, stratified for metastatic site. The top 15 up regulated and bottom 5 down regulated pathways by normalized enrichment score are shown.

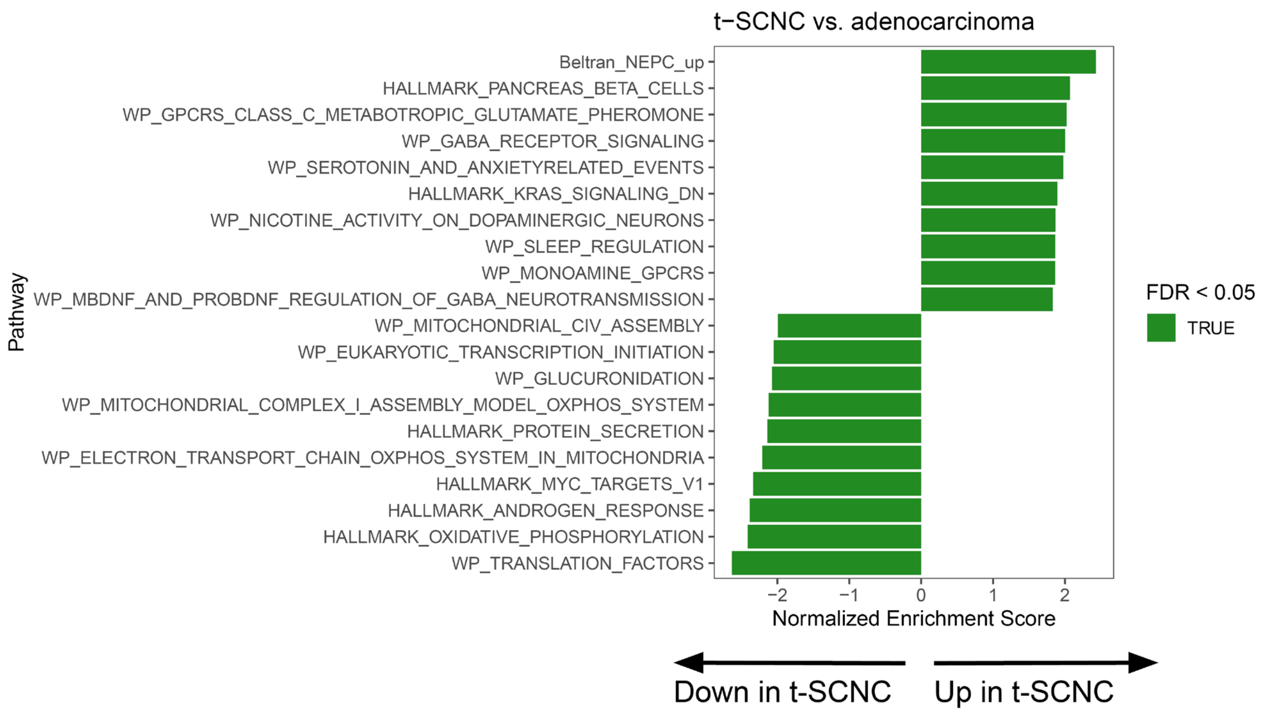

Figure S6. Differential 5hmC analysis between treatment-emergent small cell neuroendocrine prostate cancer (t-SCNC) and adenocarcinoma.

Gene set enrichment analysis of genes ranked by differential 5hmC gene body counts between cluster 1 (treatment-emergent small cell neuroendocrine cancer, N=4) and cluster 2+3 (adenocarcinoma, N=89)). The top 10 up regulated and bottom 10 down regulated pathways by normalized enrichment score are shown. t-SCNC; treatment-emergent small cell neuroendocrine cancer.

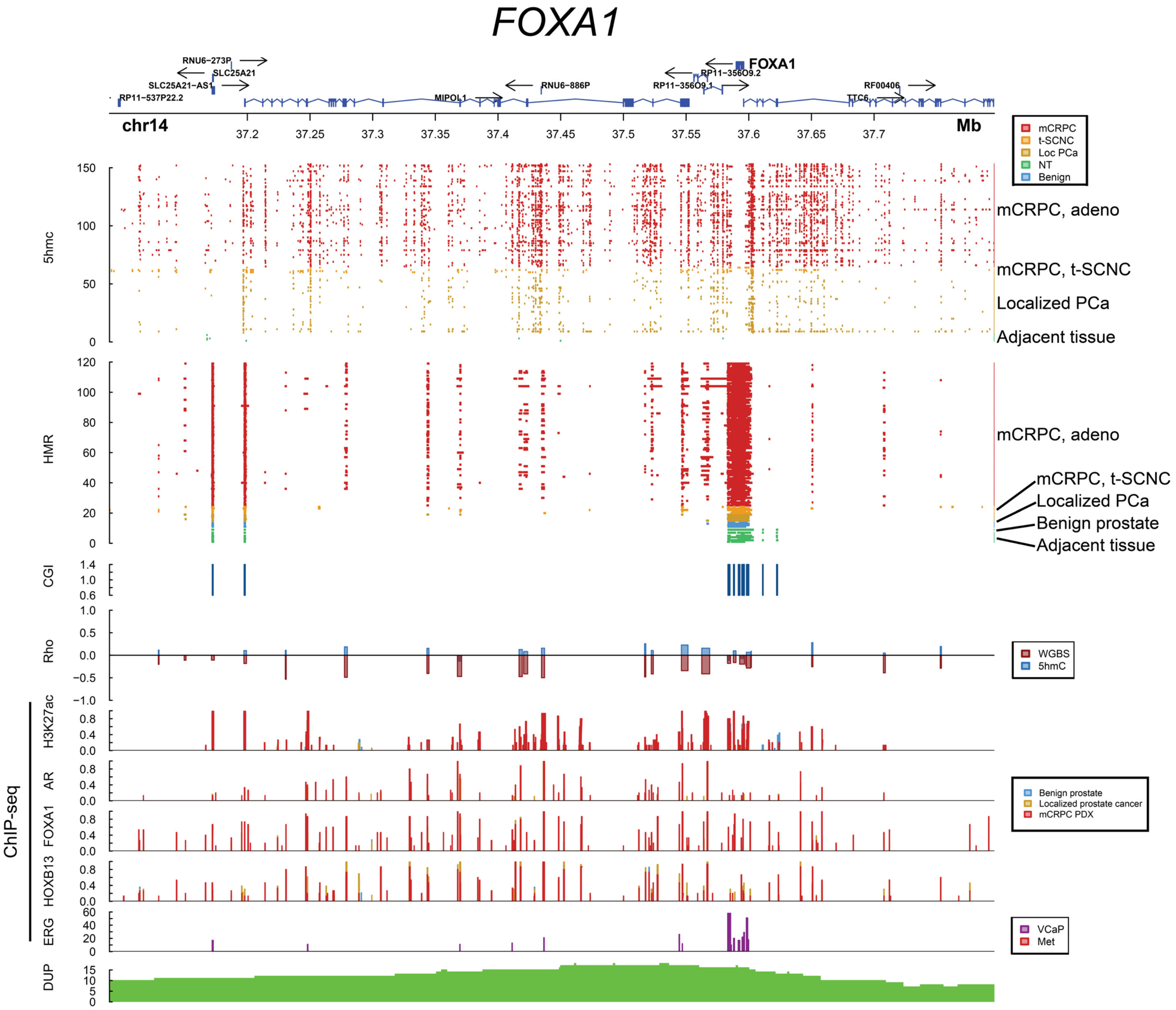

Figure S7. 5hmC marks activity of the *FOXA1* locus.
Integration of multiple layers of data for the *FOXA1* locus. 5hmC represents peaks called by MACS2 for each sample. HMR; hypomethylated regions called by whole-genome bisulfite sequencing per sample. CGI; CpG islands, Rho; Spearman's correlation between 5hmC peaks and gene expression, and methylation levels by whole-genome bisulfite sequencing, respectively, ChIP-seq; chromatin immunoprecipitation sequencing from publicly available patient samples, xenografts and cell lines. DUP; number of mCRPC samples with tandem duplications. Benign; benign prostate tissue, Localized; localized prostate cancer, mCRPC; metastatic castration-reisistant prostate cancer, NT; normal adjacent tissue (to mCRPC biopsy).

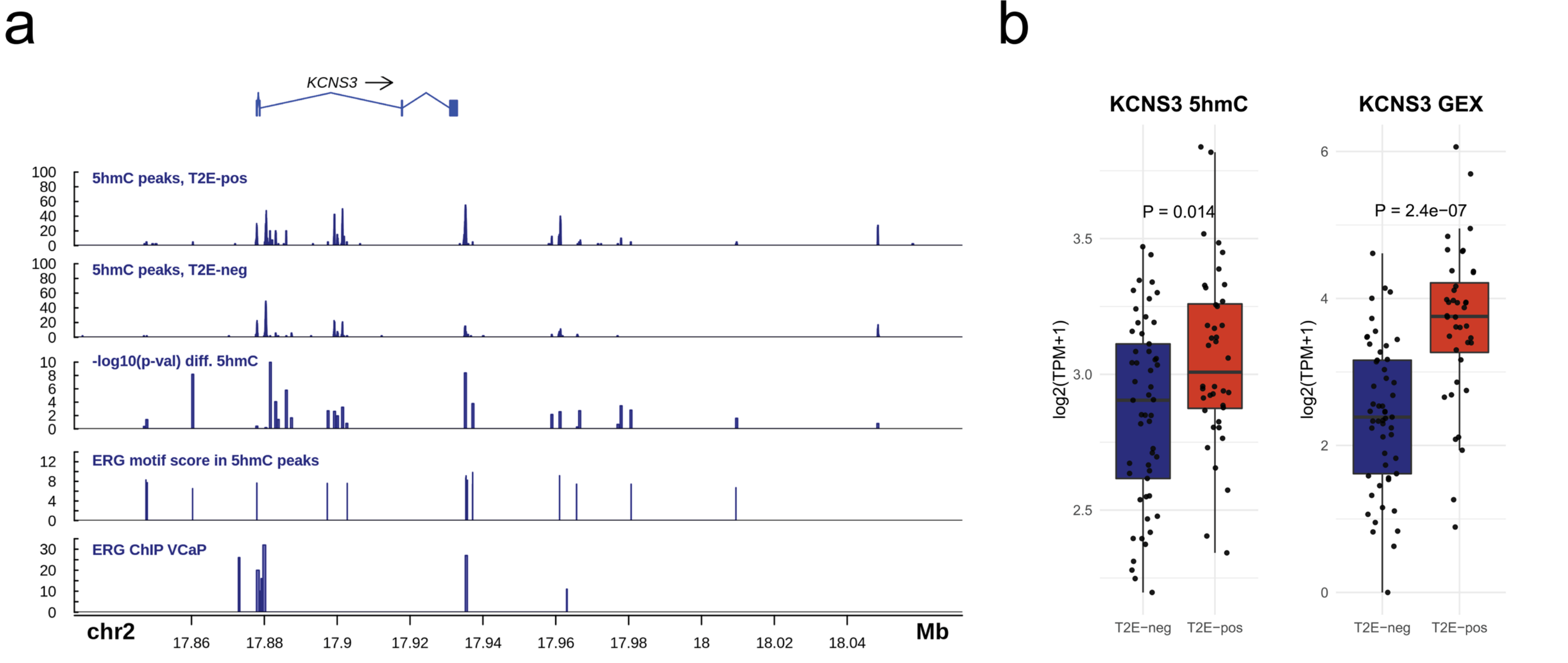

Figure S8. The *ERG*-regulated gene *KCNS3* has increased gene expression and 5hmC gene body counts and 5hmC is enriched at *ERG* binding motifs in *TMPRSS2-ERG* fusion positive samples.

a) 5hmC levels at the *KCNS3* locus. 5hmC levels are shown as frequency of samples with a peak called at every position. Samples are split by *TMPRSS2-ERG* fusion status (T2E). P-value is shown for differential 5hmC counts at consensus peaks. The motif score for *ERG* binding was calculated using HOMER for each consensus peak. **b)** 5hmC gene body counts and gene expression of *KCNS3* split by *TMPRSS2-ERG* fusion status (T2E-negative N=53, T2E positive N=40). GEX; gene expression.

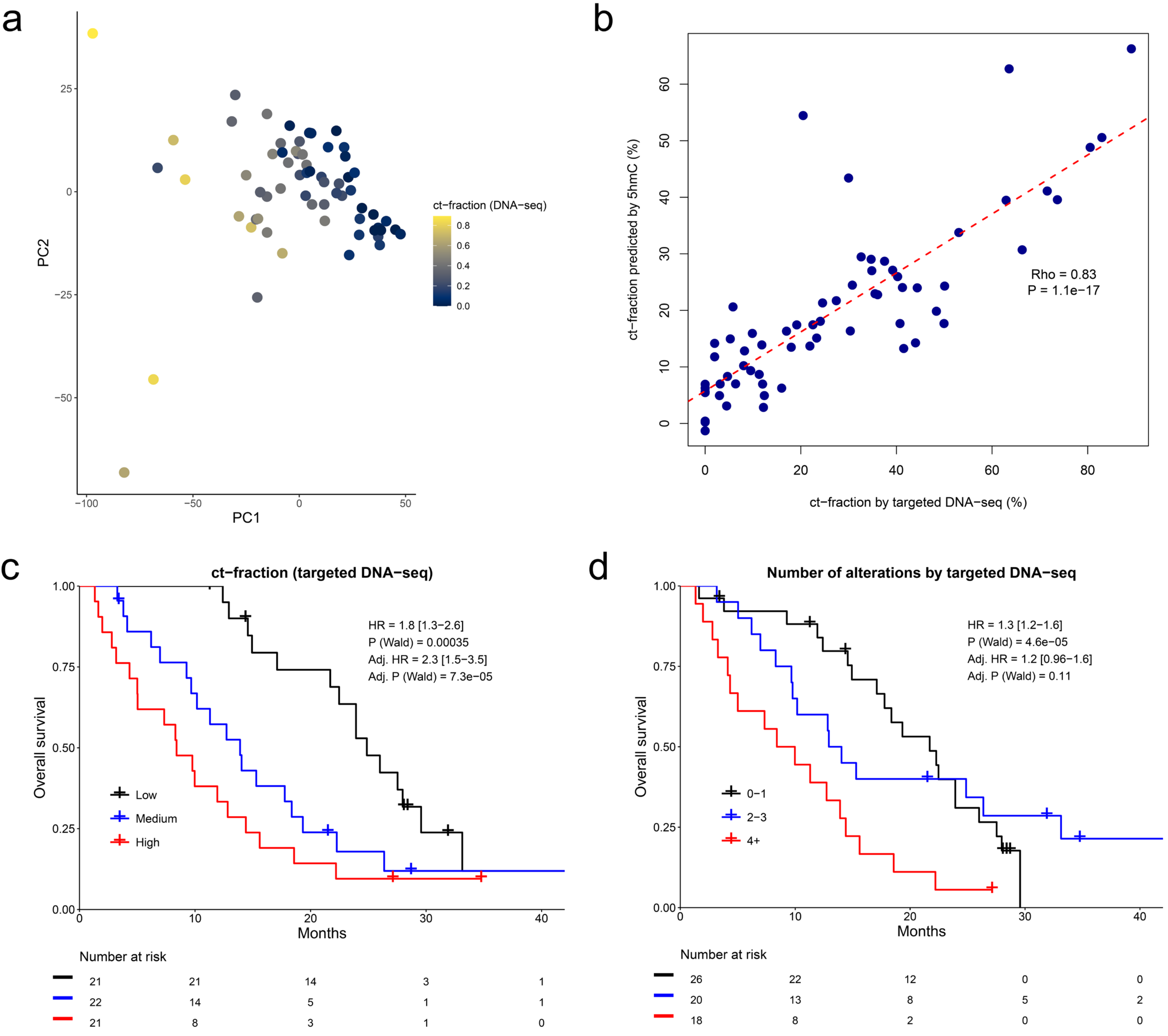

Figure S9. 5hmC patterns and targeted sequencing of cell-free DNA.
a) Principal component analysis using 5hmC gene body counts of the top 20% most variable protein coding genes. Samples are colored by the ct-fraction estimated by targeted cell-free DNA sequencing. **b)** Scatter plot of estimated ct-fraction by targeted cell-free DNA sequencing and ct-fraction predicted by a novel 5hmC classifier. c**)** Overall survival for patients split by tertiles of predicted ct-fraction from targeted cfDNA sequencing (N=64). **d)** Overall survival based on the number of genomic events inferred by targeted cell-free DNA sequencing of the eight most commonly altered genes. Alterations by targeted cell-free DNA sequencing were defined as *AR* amplification (2 or more extra copies), *MYC* gain (1 or extra more copies) and *NCOA2* gain (1 or more extra copies or a translocation), *NKX3-1* loss (at least one copy lost) *BRCA2* (at least one copy lost or inactivation by SNV or SV), *PTEN* (at least one copy lost or inactivation by SNV or SV), *TP53* (at least one copy loss or inactivation by SNV, SV or translocation) and *RB1* (at least one copy loss or inactivation by SNV or translocation). Kaplan-Meier curves were plotted for 0-1, 2-3 and >3 events, and hazard ratios were calculated as mean for each additional event inferred. P-values were calculated by Wald's test. Adjusted hazard ratios were adjusted for circulating tumor-fraction, age at mCRPC diagnosis, PSA at first-ARSI, Hb at first-line ARSI, type of ARSI (enzalutamide or abiraterone), docetaxel for metastatic hormone-sensitive prostate cancer, time to CRPC from start of ADT and presence of visceral metastases. OS; overall survival, HR; hazard ratio, ARSI; androgen receptor signaling inhibitor, ct-fraction; circulating tumor-fraction, SV; structural variant, SNV; single-nucleotide variant.

References

1. Pomerantz MM, Qiu X, Zhu Y, Takeda DY, Pan W, Baca SC*, et al.* Prostate cancer reactivates developmental epigenomic programs during metastatic progression. Nature genetics **2020**;52(8):790-9 doi 10.1038/s41588-020-0664-8.
